# Semaglutide modulates prothrombotic and atherosclerotic mechanisms, associated with epicardial fat, neutrophils and endothelial cells network

**DOI:** 10.1186/s12933-023-02096-9

**Published:** 2024-01-03

**Authors:** David García-Vega, David Sánchez-López, Gemma Rodríguez-Carnero, Rocío Villar-Taibo, Juan E. Viñuela, Adán Lestegás-Soto, Ana Seoane-Blanco, María Moure-González, Susana B. Bravo, Ángel L. Fernández, José R. González-Juanatey, Sonia Eiras

**Affiliations:** 1 Cardiology department, Complejo Hospitalario Universitario de Santiago, Travesía de la Choupana SN, 15706 Santiago de Compostela, Spain; 2 CIBERCV, ISCIII, Madrid, Spain; 3 Translational Cardiology, Health Research Institute of Santiago de Compostela (IDIS), Santiago de Compostela, Spain; 4 Present Address: Endocrinology and Nutrition Division, Complejo Hospitalario Universitario de Santiago de Compostela, Santiago de Compostela, Spain; 5 Epigenomics in Endocrinology and Nutrition Group, Epigenomics Unit, Health Research Institute of Santiago de Compostela (IDIS), Santiago de Compostela, Spain; 6 Neoplasia and Differentiation of Endocrine Cells Group, Health Research Institute of Santiago de Compostela (IDIS), Santiago de Compostela, Spain; 7 Immunology Laboratory, Complejo Hospitalario Universitario de Santiago de Compostela, Santiago de Compostela, Spain; 8 Proteomics Unit, Health Research Institute of Santiago de Compostela, Santiago de Compostela, Spain; 9 Department of Cardiac Surgery, Complexo Hospitalario Universitario de Santiago, Santiago de Compostela, Spain; 10 University of Santiago de Compostela (USC), Santiago de Compostela, Spain

**Keywords:** Semaglutide, Epicardial fat, Obesity, Atherosclerosis

## Abstract

**Background:**

Obesity has increased in recent years with consequences on diabetes and other comorbidities. Thus, 1 out of 3 diabetic patients suffers cardiovascular disease (CVD). The network among glucose, immune system, endothelium and epicardial fat has an important role on pro-inflammatory and thrombotic mechanisms of atherogenesis. Since semaglutide, long-acting glucagon like peptide 1- receptor agonist (GLP-1-RA), a glucose-lowering drug, reduces body weight, we aimed to study its effects on human epicardial fat (EAT), aortic endothelial cells and neutrophils as atherogenesis involved-cardiovascular cells.

**Methods:**

EAT and subcutaneous fat (SAT) were collected from patients undergoing cardiac surgery. Differential glucose consumption and protein cargo of fat-released exosomes, after semaglutide or/and insulin treatment were analyzed by enzymatic and TripleTOF, respectively. Human neutrophils phenotype and their adhesion to aortic endothelial cells (HAEC) or angiogenesis were analyzed by flow cytometry and functional fluorescence analysis. Immune cells and plasma protein markers were determined by flow cytometry and Luminex-multiplex on patients before and after 6 months treatment with semaglutide.

**Results:**

GLP-1 receptor was expressed on fat and neutrophils. Differential exosomes-protein cargo was identified on EAT explants after semaglutide treatment. This drug increased secretion of gelsolin, antithrombotic protein, by EAT, modulated CD11b on neutrophils, its migration and endothelial adhesion, induced by adiposity protein, FABP4, or a chemoattractant. Monocytes and neutrophils phenotype and plasma adiposity, stretch, mesothelial, fibrotic, and inflammatory markers on patients underwent semaglutide treatment for 6 months showed a 20% reduction with statistical significance on FABP4 levels and an 80% increase of neutrophils-CD88.

**Conclusion:**

Semaglutide increases endocrine activity of epicardial fat with antithrombotic properties. Moreover, this drug modulates the pro-inflammatory and atherogenic profile induced by the adiposity marker, FABP4, which is also reduced in patients after semaglutide treatment.

**Supplementary Information:**

The online version contains supplementary material available at 10.1186/s12933-023-02096-9.

## Introduction

Obesity, inflammatory and metabolic disorder, is associated with adiposopathy [1], insulin resistance (IR) [2] and cardiovascular disease (CVD) [3]. The crosstalk between adipocytes and neutrophils [4], which are increased [5]and infiltrated into adipose tissue [6] participates in this pathological condition. The released molecules by adipose tissue, named adipokines, are also good players on neutrophilia [7] and phenotypic changes of neutrophils [8], based on proteins of cell membrane and enzymes that accelerate their adhesion over endothelial cells [9] or IR in adipocytes [10], respectively. Chronic inflammation in obesity is related to higher neutrophils infiltration on adipose tissue, through interaction between neutrophils-CD11b and adipocytes-intercellular adhesion molecule 1 (ICAM-1) [6] that precede macrophages infiltration. Our previous results have also demonstrated higher CD11b expression levels on circulating neutrophils in patients with CVD and obesity than those without obesity. In fact, it was accentuated in patients with HFpEF [8],and coronary artery disease (CAD) is one of its significant risk factors [11]. Atherogenesis is one of the involved mechanisms where the role of neutrophils was underestimated due to their short life span or their phenotypic plasticity. However, important studies have demonstrated their role on early phases after atherogenic diet exposure [12]. Neutrophils, after their polarization, can migrate and adhere over vascular endothelium through the interaction CD11b and ICAM-1 [13] Afterwards, their released proteins from granules participates in the recruitment and activation of monocytes, macrophages, and dendritic cell subsets [14].Recent study, CRISP-CT, showed that the accumulation of the inflamed adipose tissue, detected by computerized tomography (CT), is a good predictor for coronaries plaques vulnerability [15]. Thus, modulation of the neutrophil’s phenotype might be considered a therapeutic target of atherosclerosis [16]. Some data, regarding glucagon-like peptide 1 (GLP-1), have demonstrated its direct and indirect mechanisms on immune system modulation [17].This molecule can mitigate the induced neutrophils markers after myocardial infarction [18]. Mainly, GLP-1 acts through a G-protein coupled receptor, that is expressed also in adipocytes [19]. This peptide stimulates the glucose-dependent insulin secretion, reduces appetite [20], modulates lipogenesis in adipose tissue cells [21] and improves their insulin sensitivity [22]. Several clinical trials with semaglutide, long-acting GLP1ra, demonstrated reduction on body weight [23] and on major accident cardiovascular events (MACE) [24]. However, the meta-analysis of the EXSCEL and FIGHT Trials showed no benefit of GLP-1ra on patients with heart failure reduced ejection fraction (HFrEF) because might increase the heart rate [25].One of the main reasons is that GLP-1 can act on sinoatrial node and parasympathetic nervous system [26]. However, the concomitant β-blockers treatment might attenuate this mechanism [27]. The early stages of HF are mainly associated with atherosclerosis as consequence of arteries stiffness, inflammation, etc.… [28]. High glucose and adipokines levels (i.e. leptin), can increase endothelial-adhesion molecules (ICAM-1, vascular cell adhesion molecule-1 (VCAM-1), and E-selectin) [29, 30]; which allows the leukocytes adhesion and transmigration through vessels. Fatty binding protein 4 (FABP4) is also an adipokine highly enhanced after adipogenesis induction in epicardial and subcutaneous fat cells [30] and their plasma levels are associated with CAD severity [31]. These results might suggest the association between higher adiposity or metabolic disorder [32, 33], represented by high plasma FABP4 levels, and CAD. This adipokine is involved on endothelial metabolism and angiogenesis [34] and adipocytes inflammation and IR [35].Their proinflammatory mediators, complement activation, and neutrophils activity might initiate an immunothrombosis pathway [36]. Although most of the GLP-1ra have demonstrated a reduction on MACE incidence, we aimed to test the role of semaglutide in this network on preclinical studies based on endocrine activity of human adipose tissue, neutrophils, and endothelial cells and plasma and cell markers modulation on treated patients for 6 months.

## Material and methods

The semaglutide (NNC 0113-217) was given by Novo-Nordisk (Bagsværd, Denmark) reconstituted and diluted according to manufacturer`s recommendations.

### Epicardial and subcutaneous fat

The protocol was approved by the Ethics Committee of Clinical Research of Galicia. Explants of epicardial adipose tissue (EAT) and subcutaneous adipose tissue (SAT) from 12 patients undergoing cardiac surgery, after signing informed consent and following the Helsinki Declaration of Ethical Principles, were taken from the upper right ventricle and thoracic region, respectively.

GLP1 receptor (GLP1R) expression was determined after RNA isolation, following the manufacturer's protocol of AllPrep DNA/RNA/Protein Mini Kit (Qiagen, Hilden, Germany). Five hundred ng were retro-transcripted, using Maxima First Strand cDNA Synthesis Kit (Thermo Fisher Scientific, Waltham, MA, USA) and 2 μl of cDNA was used for amplifying several regions of the glucagon like peptide 1 receptor (GLP1R), transcript variant 1, mRNA (NM_002062.5); GLP1R_a (F:5′-CAAATGCAGACTTGCCAAGTCCACG-3′;R:5′-CAGCTGGACCTCATTGTTGACAAAG-3′),GLP1R_b(F:5′-CACCTCCTTCCAGGGGCTGATGG-3′;R:5′ TCAGGCTGCTGGTGGGACACTTG-3′) and GLP1R_n (F:5′-TACTGCATGAGCAGAAACACC-3′; R:5′-GAACCTGTTTGCATCCTTCATC-3′), and b-actin (ACTB) mRNA (NM_001101.5; F:5’- TTCTGACCCATGCCCACCAT-3’; R: 5’-ATGGATGATGATATCGCCGCGCTC-3’). These primers were amplified by quantitative polymerase chain reaction using the FastStart SYBR Green Master (Hoffman-La Roche, Basel, Switzerland) at 40 cycles (95 °C for 30 s, 60 °C for 60 s and 72 °C for 30 s) in a QuantStudio 3 (Thermo Fisher Scientific). The cycle threshold (Ct) values of the genes were normalized by the Ct values of ACTB (ΔCt). The differential expression levels were represented as arbitrary units (a.u.) based on 2-(ACTB/gene) algorithm.

Biopsies of ~ 250 mg were taken immediately after pericardiotomy and transported to the laboratory in sterile conditions within 30 min and washed with sterile saline solution for 60 min at 4 ºC. Afterwards, in sterile conditions, they were split in equal pieces (50 mg/each), cultured in medium M199 with Earle’s Salts (0.25 mL) that contains glucose 100 mg/dL and treated with semaglutide (1 nM), insulin (2.5 or 5 μg/mL; Sigma-Aldrich Co., St Louis, MO,USA), or both for 90 min. The final groups of treatments were control, insulin (INS), semaglutide (Sema), insulin and semaglutide (Sema + INS) treatment. At the end of the treatment, supernatants were collected and used for glucose measurements, by colorimetric assay kit (Cayman Chemical, Michigan, USA), and exosomes isolation, by Exo-Spin Exosome Purification Kit (Cell Guidance Systems, Cambridge, UK) according to the manufacturer’s protocol. Isolated exosomes were concentrated 3X using Amicon Ultra 0.5 mL 3 k Millipore’s columns (Merck Millipore Ltd., Cork, Ireland). Validation of isolated exosomes was performed by specific western blot. Proteins were denaturalized with Laemmli buffer (5X) at 95 ºC for 5 min. Then, they were loaded and separated in a 10 or 12% SDS-PAGE using vertical electrophoresis and following buffer (250 mM Tris-Base, 1.92 M Glycine, 0.5% SDS) at 40 mA for 120 min. At the end, separated proteins were transferred to fluorescence-adapted polyvinylidene difluoride (PVDF) membrane (Immobilon FL-Membrane, Merck Millipore) using the following buffer (25 mM Tris-Base, 192 mM glycine, 0.0125% SDS and 20% methanol) at 400 mA for 60 min. Afterwards, membrane was washed, blocked with 5% bovine albumin serum for 60 min and incubated with primary mouse monoclonal antibodies (mAb): mAb anti-CD81 (1 μg/mL), mAb anti-CD63 (1 μg/mL), mAb anti-CD9 (0.5 μg/mL; Exosome Detection Antibody Pack, Novus Biologicals, Bio-Techne, Minneapolis, EEUU) and rabbit mAb anti-FABP4 (1 μg/mL; Invitrogen, Life Technologies Corp., Waltham, MA USA) and secondary antibodies anti-mouse Alexa Fluor 488 (2 μg/mL) and anti-rabbit Alexa Fluor 532 (2 μg/mL; Invitrogen). Visualized proteins, as bands, were detected by fluorescence detector ChemiDoc MP (Bio-Rad) and quantified by ImageLab™ software (6.1.0 build 7 Standard Edition, Bio-Rad).

### Proteomics of released exosomes

Isolated exosomes from treated EAT and SAT were concentrated 3X using Amicon Ultra 0.5 mL 3 k Millipore’s columns (Merck Millipore Ltd). After trypsin digestion, proteins were identified by mass spectrometry. Peptides were separated using Reverse Phase Chromatography. Gradient was developed using a micro liquid chromatography system (Eksigent Technologies nanoLC 400, SCIEX Foster City, USA) coupled to high-speed Triple TOF 6600 mass spectrometers (SCIEX Foster City, USA) with a micro flow source. The analytical column used was a silica-based reversed phase column Chrom XP C18 150 mm × 0.30 mm, 3 mm particle size and 120 Å pore size (Eksigen, Dublin, CA, USA). The trap column was a YMC-TRIART C18 (YMC Technologies, Teknokroma) with a 3 mm particle size and 120 Å pore size, switched online with the analytical column. The loading pump delivered a solution of 0.1% formic acid in water at 10 μl/min. The micro pump provided a flow-rate of 5 μl/min and was operated under gradient elution conditions, using 0.1% formic acid in water as mobile phase A, and 0.1% formic acid in acetonitrile as mobile phase B. Peptides were separated using a 90 min gradient ranging from 2 to 90% mobile phase B (mobile phase A: 2% acetonitrile, 0.1% formic acid; mobile phase B: 100% acetonitrile, 0.1% formic acid). Injection volume was 4 μl. Data acquisition was carried out in a Triple TOF 6600 System (SCIEX, Foster City, USA) using a data dependent workflow. Source and interface conditions were as follows: ion spray voltage floating (ISVF) 5500 V, curtain gas (CUR) 25, collision energy (CE) 10 and ion source gas 1 (GS1) 25. Instrument was operated with Analyst TF 1.7.1 software (SCIEX). Switching criteria was set to ions greater than mass to charge ratio (m/z) 350 and smaller than m/z 1400 with charge state of 2–5, mass tolerance 250 ppm and an abundance threshold of more than 200 counts (cps). Former target ions were excluded for 15 s. Instrument was automatically calibrated every 4 h using tryptic peptides from pepcalmix as external calibrant. After MS/MS analysis, data files were processed using Protein Pilot TM 5.0.1 software from Sciex, which uses the algorithm ParagonTM for database search and ProgroupTM for data grouping. Data were searched using a Human specific Uniprot database. False discovery rate was performed using a non-lineal fitting method displaying only those results that reported ≥ 1% global false discovery rate [37]. All identified proteins at least in 2/3 samples were considered for differential analysis by FunRich (version 3.1.3). The best network between those differential proteins among treatments (control, INS, sema or sema + INS) and insulin or GLP1R signalling was performed using STRING database [38] or Uniprot [39].

### Neutrophils (HL-60 cells)

Same number (1 × 10^6^) of promyelocytic human cell line HL-60, seeded on 25 cm^3^ flask (Corning, Corning, NY, USA) was differentiated into neutrophils (dHL-60) with 1.26% dimethyl sulfoxide (DMSO) (Sigma-Aldrich) in RPMI (Gibco, Life Technologies Limited, Paisley, UK) for 6 days.

GLP1R and CD11B expression was determined after RNA isolation, following the manufacturer's protocol of AllPrep DNA/RNA/Protein Mini Kit (Qiagen, Hilden, Germany). Five hundred ng were retro-transcripted, using Maxima First Strand cDNA Synthesis Kit (Thermo Fisher Scientific) and 2 μl of cDNA was used for amplifying CD11B (F:5′-CAGCCTTTGACCTTATGTCATGG-3′; R:5′-CCTGTGCTGTAGTCGCACT-3′) and GLP1R, as it was described before. Afterwards, dHL-60 were seeded on a 24-well plate (3–5 × 10^5^ /well)(NEST BioTechnologies Co., Wuxi, China) with RPMI and treated with N-formylmethionyl-leucyl-phenylalanine (fMLP) (Sigma-Aldrich Co) at 10 μM or FABP4 (CloudClone Corp., Wuhan, China) at at 6.8 nM for 90 min under or not semaglutide (1 nM) presence or pretreatment. Adhesion marker, CD11b, was analyzed by flow cytometry. Cells were incubated with specific antibody CD11b (CD11b-PE; BD Biosciences, Becton–Dickinson, Franklin Lakes, USA) at room temperature for 45 min. At the end, cells were washed with FACS flow (BD Biosciences). After centrifuging cells at 350xg for 7 min, they were resuspended in 1 mL of 1% paraformaldehyde (PFA) (Thermo Fisher Scientific). Fluorescence was detected by Axio Vert.A1 (ZEISS, Oberkochen, Germany) or flow cytometer FACSCalibur (BD Biosciences) and analyzed with Flow Jo™ Software (BD Biosciences). Cells were gated on their characteristic forward (FSC) and side scatter (SSC) after 10000 events acquisition. CD11b was represented as relative fluorescence units (RFU).

We also measured the myeloid marker CD11b, by immunocytochemistry (ICC) according to the protocol described by Tsang et al., 2017 [40]. Briefly, 1 × 10^6^ of dHL-60 cells were seeded in a 24-well plate and treated with fMLP at 10 μM or its vehicle without serum for 90 min. Afterwards, RPMI medium was removed carefully, and cells were washed with sterile saline solution. Then, cells were fixed with 4% PFA for 15 min and permeabilized with 0.5% Triton X-100 (Sigma-Aldrich Co.) for 10 min. After washing cells were blocked with 1% BSA (Sigma-Aldrich Co.) at 37 ºC for 30 min. Finally, cells were incubated with CD11b-PE antibody (BD Biosciences) in 1% BSA for 45 min, counterstained with NucBlue™ Fixed Cell ReadyProbes™ (Thermo Fisher) and visualized with fluorescence microscope Axio Vert A1 (ZEISS).

Migration activity of dHL-60 was performed using a Sun-Chip (B-flow, Santiago de Compostela, Spain). The chip was filled with medium and cells (5 × 10^4^), stained with green calcein-AM (Invitrogen) at 1 μM for 20 min, were included on the core or side holes of Sun-chip. After including 10 μL of fMLP 50 mM (Sigma-Aldrich Co.), as chemoattractant, fluorescence tracer was registered for 20 min using Chemidoc MP Imaging equipment (BioRad). These data were analyzed using ImageJ software [41].

### Human aortic endothelial cells (HAEC)

Human Aortic Endothelial Cells (HAEC) were purchased from Lonza (Lonza Bioscience, Basel, Switzerland) and cultured according to manufacturer’s recommendations using M199 with Earle’s salts (Gibco) and supplemented with 10% foetal bovine serum (FBS) (Gibco). After subculturing, cells were seeded in 24-well plates (NEST BioTechnologies Co.) until reach 95% confluence. After staining with Cell Tracking Red Dye Kit (Abcam, Cambridge, UK) for 24 h, monolayer was exposed to normal (NG; 5.55 mM) or high glucose concentrations (HG; 22.2 mM; Sigma-Aldrich Co.) in M199 without FBS for 30, 120 or 360 min. Then, treated dHL-60 (2 × 10^5^ cells/well) with fMLP at 10 μM (Sigma Aldrich Co.) and/or semaglutide in RPMI for 90 min were stained, washed, resuspended with M199, and incubated for 1 h with endothelial monolayer, pretreated or not with HG. The non-attached neutrophils were washed with saline solution. Finally, fluorescence was detected by ChemiDoc Imaging system and microscopy after being fixed with 4% PFA for 15 min. Adhered neutrophils, based on green stained cells, and total number of endothelial cells, based on stained nuclei with DAPI, were calculated using ImageJ. Ratio between adhered neutrophils and total endothelial cells number was represented.

### Angiogenesis assay

We evaluated angiogenic capacity of HAEC under semaglutide at 1 nM, fat-released protein, FABP4 at 6.8 nM (CloudClone Corp), or both treatments using angiogenesis assay kit (Abcam) according to manufacturer’s protocol. Twenty thousand cells were added to precoated 96-well plates with extracellular matrix gel and treatments at 37 ºC and 5% CO2 for 20 h. Images, after staining, were visualized using AxioVert A1 fluorescence microscope and quantified by Image J. Number and percentage of covered area by meshes was calculated using angiogenesis analyzer plug-in [42].

### Markers and Semaglutide treatment on patients

We performed an observational and longitudinal study in patients who in clinical practice started treatment with semaglutide. Following the ethical principles from Declaration of Helsinki and committee approval from our institution (Clinical Research Committee Ethics of Galicia, Spain). Inclusion criteria were type 2 diabetes mellitus (T2DM), BMI ≥ 30 kg/m2 and age ≥ 18 years old with poor glycaemic control (Hb1AC ≥ 7%), despite 2 antidiabetic drugs. The main exclusion criteria were type 1 diabetes, other prescribed GLP-1ra, any active infection, pregnancy, cancer or chronic kidney disease on haemodialysis. Subcutaneous semaglutide was titrated up to maintenance dose (0.5 or 1.0 mg once weekly), after signing the consent form. Visits were programmed as follow: Day 1 (inclusion), day 30 (up titration) and 6 months (follow up visit). Inclusion visit (day 1) consisted in explanation and signing duplicate consent form, medical record, physical exploration determining vital signs, weight, high, waist, hip, arm and thigh ratios, 12 derivation electrocardiogram, transthoracic echocardiography and body composition was determined by bioelectrical impedance analysis using InBody test (InBody 770, Tokyo, Japan) according to manufacturer’s protocol. The main considered measures were visceral fat area (cm^2^), lean mass, fat mass (kg) and skeletal muscle mass (kg). Fasting blood samples (EDTA, citrate and heparin tube, 5 mL each one) were obtained for routine laboratory measuring (hemogram, coagulation and biochemistry) and research analysis of neutrophils and monocytes phenotype and additional plasma protein markers was done on translational cardiology laboratory. We performed a 30-day visit to evaluate semaglutide tolerance and up titration. After 6 months, the protocol was repeat during the routine visit, medical record, fasting blood samples, physical exploration, electrocardiogram, echocardiography and InBody test. Unscheduled face-to-face or telephonic consultations were done if needed.

Fasting blood samples into EDTA coated vacutainers (BD-Plymouth, UK) were transferred and processed within the first hour of extraction for neutrophils and monocytes phenotype analysis. Total blood, 120 μL, was used for monotytes characterization: CD16 (FITC Mouse Anti-Human CD16, BD Pharmingen™, BD Biosciences), CD14 (APC Mouse Anti-Human CD14, BD Pharmingen™, BD Biosciences), C–C chemokine receptor type 5 (3.75 μg/mL; PE-CCR5; Biolegend, San Diego, CA, USA) and HLA-DR (0.78 μg/mL; Anti-HLA-DR PerCP, BD™, BD Biosciences) or neutrophils: CD11b (PE Mouse Anti-Human CD11b, BD Pharmingen™, BD Biosciences), CD88 (3.75 μg/mL;APC anti-human CD88 [C5aR] Antibody, Biolegend), CXCR2 (15 μg/mL; FITC anti-human CD182 [CXCR2] Antibody, Biolegend) and HLA-DR (Anti-HLA-DR PerCP, BD™, BD Biosciences). Antibodies were incubated for 45 min. After, erythrocytes were lysed with 2 mL of 1X BD FACS lysing solution (BD Biosciences) during 10 min. After centrifuging at 350xg for 7 min, cells were resuspended in 1 mL FACS Flow (BD Biosciences) and measured by flow cytometer FACSCalibur (BD Biosciences), using the Flow Jo™ Software (Becton Dickinson and Company).

We have selected different neutrophils (CD11b, CXCR2 and CD88) and monocytes phenotype markers (CD14, CD16, CCR5). The neutrophils molecules are related to endothelial adhesion, maturation and migration [43, 44]. The monocytes markers can distinguish classical (CD14^+^CD16^−^), intermediates (CD14^+^CD16^+^), and non-classical (CD14^−^CD16^+^), monocytes, being CCR5 higher in intermediates and pro-inflammatory monocytes [45]. We also selected plasma protein biomarkers based on adiposity and metabolic markers (FABP4, leptin, insulin), ligands of CXCR2 or CD88, IL-8 and component C5a, respectively, atrial or ventricle stretch (natriuretic peptides), inflammation and fibrosis (GDF15, TSP2, IGFBP-7) [46], endothelial dysfunction (ICAM-1), and mesothelin (mesothelial marker).

Plasma proteins markers were analyzed by Luminex Discovery Assay (Biotechne). To perform the assay, samples were thawed and diluted 1:2 in Calibrator Diluent RD6-52 (Bio-Techne). The protocol consisted in three main steps (a) addition of analyte to specific magnetic microparticles (b) addition of specific biotinylated antibodies and (c) streptavidin–phycoerythrin (PE). Fluorescence was measured by Bio-Plex 200 (Bio-Rad). The device uses one laser to excite the dyes inside each microparticle to identify the microparticle region and the second laser to excite the PE to measure the amount of analyte bound to the microparticle. All the fluorescence emissions from each microparticle are analysed using a photomultiplier tube and a photodiode.

### Statistical analysis

Continuous data was checked for normality using Shapiro–Wilk test and expressed as mean ± standard deviation (SD). Mean ranks of glucose consumption were compared between each treatment and control by non-parametric Mann–Whitney test because the low amount of tissue did not allow to perform the same number of treatments per patient. The comparisons among groups of treatments on cells were performed using an ANOVA and a post-hoc Tukey’s multiple comparison test for normal distributed data. Scattered data and comparison among groups with small sample size were analysed by Friedman test and a post-hoc Dunn’s multiple comparison test. Comparisons between two treatments on cells were made using paired Student´s t test in parametric data and non-parametric test, Wilcoxon`s singed ranked tests, in skewed data and markers comparison after semaglutide treatment. Correlation between cells number and detected fluorescence was performed by linear regression analysis. Fold change of markers levels on patients after semaglutide treatment was calculated by ratio between 6 months/basal markers levels. Differences between markers regarding risk factors, CVD or treatments were performed by Mann–Whitney test. Statistical differences of marker levels before and after treatment were done by Wilcoxon test. GraphPad Prism 8.0.2 (GraphPad Software, San Diego, USA, www.graphpad.com) or SPSS Statistics for Windows, version 25.0 (SPSS Inc., Chicago, Ill., USA) were used for statistical analysis, considering significance *p < 0.05, **p < 0.01 and ***p < 0.001.

## Results

### Epicardial and subcutaneous fat

#### Endocrine activity

**Fig. 1:**
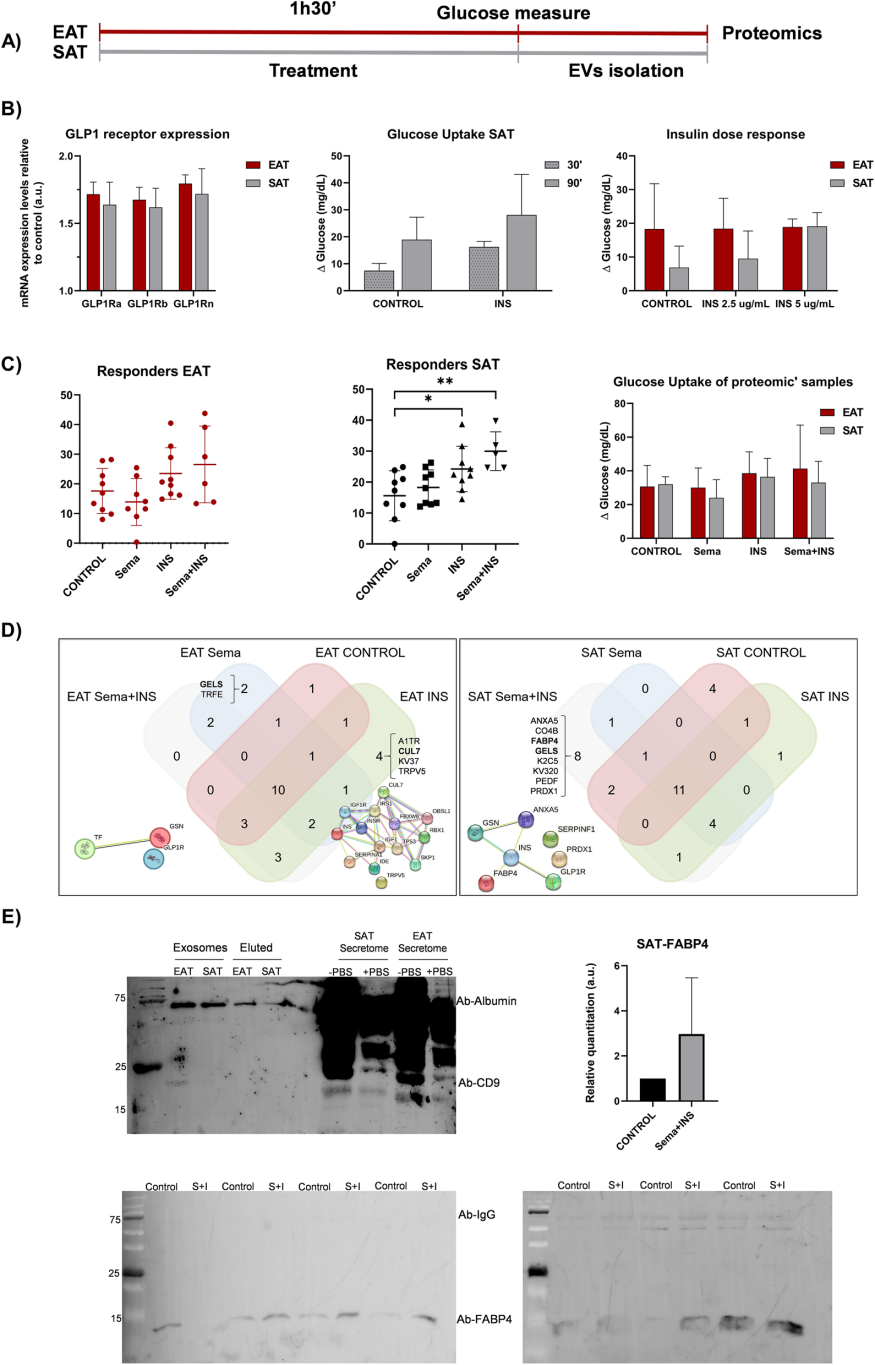
Glucose or endocrine activity of adipose tissue after Semaglutide treatment. **A** Workflow of EAT and SAT treatment with semaglutide (Sema) (1 nM), insulin (INS) (2.5 or 5 μg/mL) or both for 90 min. Afterwards, glucose was analyzed by colorimetric assay or exosomes were isolated by ExoSpin kit for proteomics analysis. **B** Bar plot shows mean ± SD of GLP1 receptor (NM_002062.5) mRNA expression levels on epicardial and subcutaneous adipose tissue (EAT and SAT) from patients underwent cardiac surgery (n = 2). Their mRNA expression levels were quantified by quantitative PCR and represented according to β-actin (NM_001101.5) expression levels as arbitrary units (a.u.) based on 2^−(ACTB/gene)^ algorithm. Different regions were checked. Bar plots shows mean ± SD of glucose uptake by EAT and SAT under different treatments and times. Glucose consumption by SAT after insulin treatment was performed for 30 and 90 min (n = 2), glucose consumption by EAT and SAT from 3 patients was analyzed after insulin treatment at different concentrations (2.5 and 5 μg/mL). Wilcoxon rank test determined unsignificant changes. **C** 9 out of 12 SAT or EAT samples were insulin responders. Dot plot shows individual data, mean ± SD of glucose consumption by EAT and SAT after semaglutide (Sema), insulin (INS) or combined treatment (Sema + INS). Mann–Whitney test did not show statistical significance regarding EAT. Nonetheless, the glucose consumption increased after insulin [n = 9, CONTROL (15.590 ± 8.053) *vs* INS (24.240 ± 7.332), *p = .0400] and combined treatment [n = 5, CONTROL (15.590 ± 8.053) *vs* Sema + INS (29.992 ± 6.243), **p = .0040]. Bar plot represents mean ± SD of glucose consumption (mg/dL) after fat biopsies treatment in those selected patients for proteomics analysis (n = 3). **D** Venn’s diagram and String plots of the identified proteins from exosomes by TripleTOF. Proteins on each group of treatment were selected if they were identified at least twice. They were included on FunRich software for identifying those differential proteins regarding treatments. A1TR (SERPINA1), CUL7, KV37 and TRPV5 were only identified after insulin (INS) treatment on EAT. STRING analysis shows a network among A1TR, CUL7 and INS. GELS (GSN) and TFRE were identified after Semaglutide (Sema) treatment. STRING analysis showed relationship with GLP1R. On subcutaneous fat (SAT), PEDF, CO4B, K2C5, KV320, PRDX1, ANXA5, GELS (GSN) and FABP4 were only identified after cotreatment (Sema and INS). STRING analysis shows a network among the last three proteins (ANXA5, GSN and FABP4), INS and GLP1R. **E** Upper left representative western blot of epicardial (EAT) and subcutaneous fat (SAT) proteins from exosomes and eluted part or total secretome with (+) or without (−) washing with saline solution (PBS) and stained with albumin and CD9 tetraspanin antibodies. Upper right bar graph represents mean ± SD of FABP4 intensity identified on following western blots of SAT with combined treatment (Sema + INS) regarding control from 7 patients. Wilcoxon test did not show statistical significance. Down representative western blots of SAT with FABP4 or IgG antibodies (Ab)

After testing and validating the GLP1R expression levels on fat biopsies from patients undergoing cardiac surgery (Fig. 1A), explants were or not treated with insulin, semaglutide or both for 30 or 90 min (Fig. 1B). Our data showed higher glucose consumption after insulin treatment for 90 min. This dose and time were selected for performing the acute treatments. Nine out of 12 tested patients (75%) were insulin responders on EAT or SAT samples (Fig. 1C). After selecting those responders, our results showed a glucose consumption difference with statistical significance between control and insulin or combined semaglutide treatment in SAT samples. Although an increment was also detected on EAT, it did not reach the statistical significance. Afterwards, we selected three patients with higher insulin response on EAT and released exosomes from biopsies were isolated and validated by western blot with specific tetraspanins antibodies (CD9, CD63 or CD81). Our results showed that CD9 antibody was able to stain a band of 23 kDa (Fig. 1D), mainly on released exosomes by epicardial fat with or without previous saline solution washed at 4ºC for 60 min. This procedure allowed a higher sample ‘purification from blood contamination. Thus, CD9 protein was not detected in non-exosomes (eluted) part (Fig. 1D). However, after studying the proteomics profile on EAT supernatants-exosomes and selecting the proteins, which were identified at least twice, cullin 7 (CUL7), alpha 1 anti-trypsin (A1TR), transient receptor potential cation channel subfamily V member 5 (TRPV5) and Probable non-functional immunoglobulin kappa variable 3–7 (KV37) were observed after insulin treatment (Fig. 1E). Vesiclepedia showed CUL7 and TRPV5 presence on serum exosomes. The String functional and association of proteins confirmed the network between insulin signal transduction and CUL7, which is an E3 ubiquitin ligase. These results validated the insulin response on tested EAT. The differential identified proteins on released exosomes after semaglutide treatment were gelsolin (GELS or GSN) and transferrin (TRFE). Regarding SAT-released exosomes, any differential protein was identified after insulin treatment. In these samples less glucose consumption was observed after insulin treatment. However, their cotreatment with semaglutide increased the identification of GELS on released exosomes. Other identified proteins were complement C4-B (CO4B), pigment epithelium-derived factor (PEDF), peroxiredoxin-1 (PRDX1), keratin type II cytoskeletal 5 (K2C5), immunoglobulin kappa variable 3–20 (KV320), FABP4 and annexin A5 (ANXA5). These last two proteins were registered in vesiclepedia and String database showed their network with insulin and GLP1 signalling (Fig. 1E).

#### Fat-released molecules and neutrophils-CD11b or angiogenesis assay

**Fig. 2:**
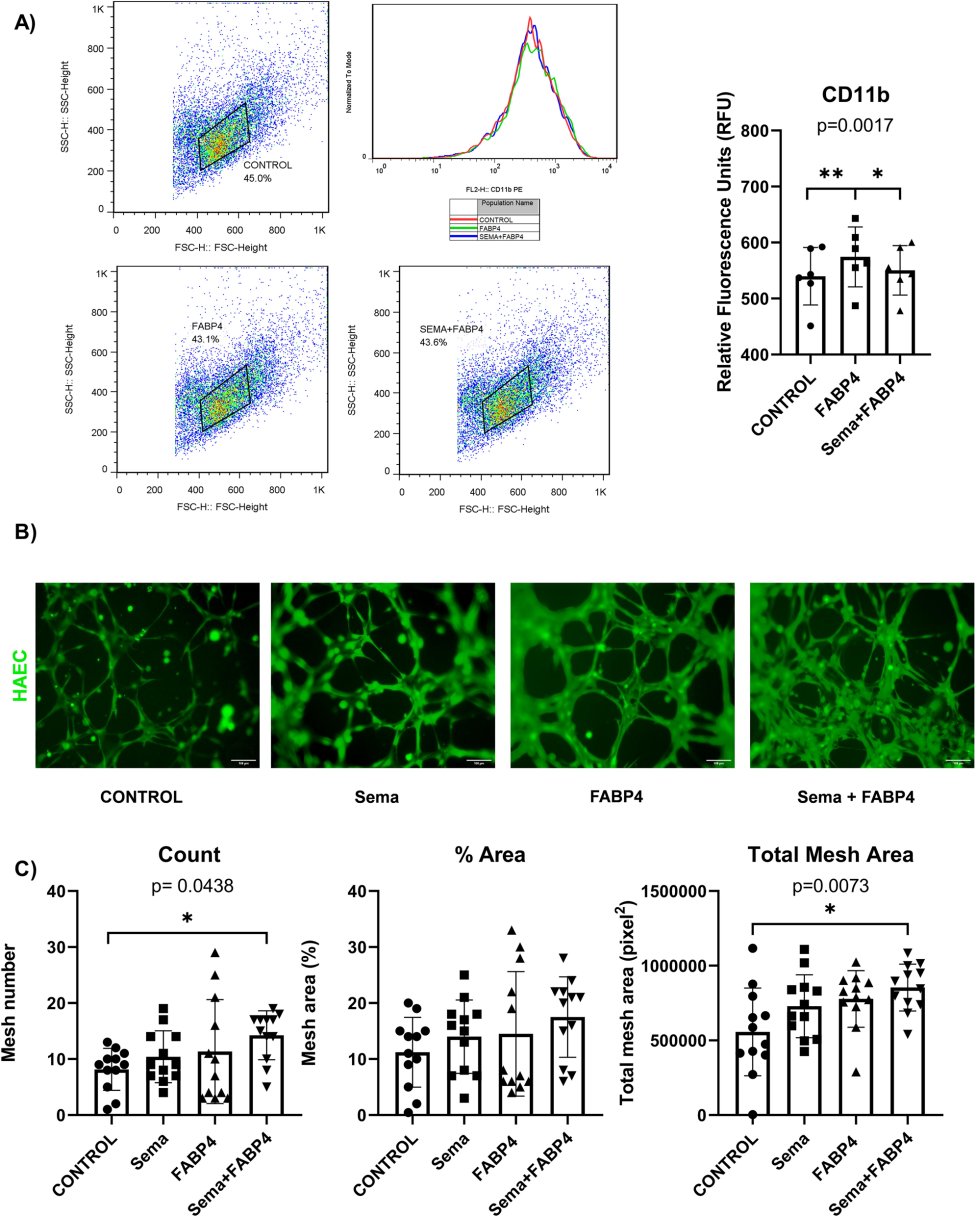
FABP4 on CD11b and angiogenesis and their regulation by Semaglutide. **A** Dot plots or histogram of CD11b expression levels, expressed by relative fluorescence units (RFU) and analyzed by flow cytometry, after FABP4 (6.8 nM) with or without semaglutide (Sema) 1 nM for 90 min on neutrophil-like dHL-60 cells. Bar graph with individual data points represents the CD11b levels after treatments. Statistical analysis shows an increase of CD11b after FABP4 treatment and modulated by semaglutide cotreatment (1 nM) [n = 6; one-way ANOVA, F(2,5) = 18.72, *p* = 0.0017; p < 0.01; Tukey’s post-hoc Control (539.8 ± 51.41) *vs* FABP4 (574.3 ± 53.41), adjusted *p* value = 0.0071; p < 0.001; Tukey’s post-hoc FABP4 (574.3 ± 53.41) *vs* Sema + FABP4 (550.3 ± 44), adjusted *p* value = 0.0345; p < 0.05]. **B** Representative fluorescence microscopy images of human aortic endothelial cells (HAEC) after treatments (semaglutide (1 nM), FABP4 (6.8 nM) or both) for 20 h on an angiogenesis assay (HAEC are shown in green. Scale bar = 100 μm). Bar graphs with individual data points represents the mesh number, mesh % over total area and total mesh area. Statistical analysis shows an angiogenesis effect of cotreatment based on number of meshes [n = 12; Friedman test, *p* = 0.043; p < 0.05; Dunn`s multiple comparisons test: Vehicle (Veh) (8.167 ± 3.738) *vs* Sema + FABP4 (14.25 ± 4.372), adjusted *p* value = 0.0339; p < 0.05] and total mesh area [n = 12 one-way ANOVA, *F*(3,11) = 3.049, *p* = 0.0064; p < 0.01; Tukey’s post-hoc Veh (557229 ± 293903) *vs* Sema + FABP4 (853809 ± 157386), adjusted *p* value = 0.0353; p < 0.05]

FABP4 is an adipocyte [47], inflammatory [48] and proangiogenic [49] marker. Our results showed that CD11b was upregulated in neutrophils dHL60 after FABP4 treatment (574.3 ± 53.41 *vs* 539.8 ± 51.41 RFU, p < 0.01) and modulated by semaglutide treatment (550 ± 44 *vs* 574.3 ± 53.41, p < 0.05 RFU) (Fig. 2A). Although angiogenesis was increased after semaglutide or FABP4 treatment, only cotreatment reached the statistical significance, measured by number (n = 12; Friedman test, p = 0.043; p < 0.05; Dunn's multiple comparisons test: Control (8.167 ± 3.738) *vs* Sema + FABP4 (14.25 ± 4.372), p adjusted value = 0.0339; p < 0.05) and total mesh area (n = 12; one-way ANOVA, F(3.11) = 3.049, p = 0.0064; p < 0.01; Tukey's post-hoc test Control (557229 ± 293903) *vs* Sema + FABP4 (853809 ± 157386), p adjusted value = 0.0353; p < 0.05) (Fig. 2B, C).

### Neutrophils and endothelial cells adhesion

**Fig. 3:**
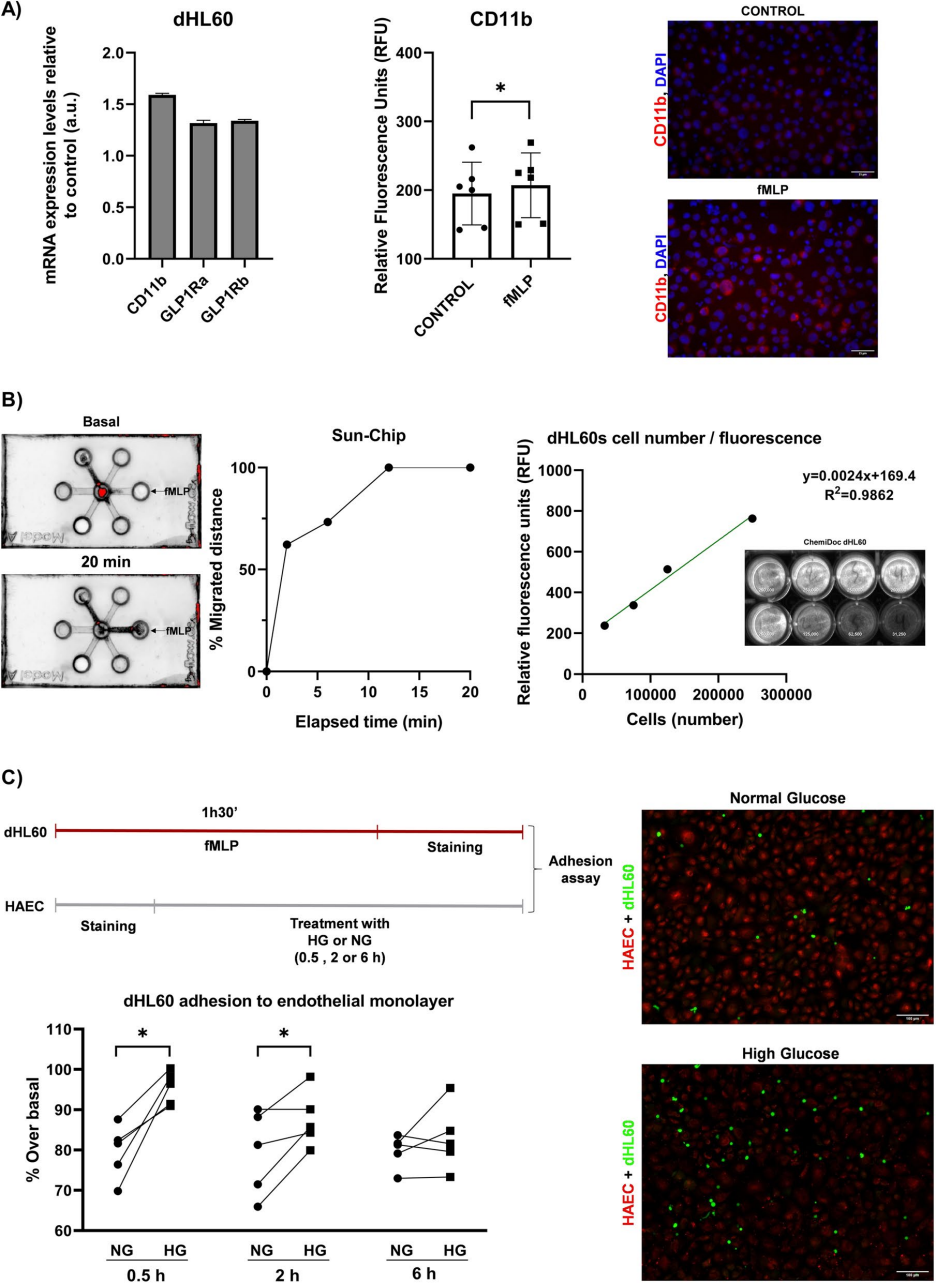
Neutrophil-like dHL-60 and adhesion to endothelial monolayer. **A** Left bar plot shows mean ± SD of GLP1 receptor and CD11b myeloid maker mRNA expression levels on neutrophil-like dHL-60 cells. Their mRNA expression levels were quantified by real time PCR and represented according to β-actin expression levels as arbitrary units (a.u.) based on 2-(ACTB/gene) algorithm. Different regions of the GLP1R (NM_002062.5) were checked. Right bar plot with individual points represents the CD11b levels on dHL-60, with/without fMLP treatment at 10 μM for 90 min, by flow cytometry, expressed in relative fluorescence units (RFU). The CD11b levels were upregulated with statistical significance after fMLP treatment [n = 6, CONTROL (195 ± 45.53) *vs* fMLP (207 ± 47.24); t(5) = 3.985, *p* = 0.0105; p < 0.05]. We also checked this upregulation by performing an immunocytofluorescence using an anti-human CD11b-PE antibody (Scale bar = 25 μm). **B** Neutrophil-like dHL-60 cells migration towards fMLP stimulus on a Sun-Chip. The neutrophils tracer and migration were detected by a ChemiDoc MP Imaging System after their fluorescence staining with calcein-AM. This method was validated for analysing the correlation between relative fluorescence units (R.F.U.) and number of cells [*R*^*2*^ = 0.9862, *F*(1,2) = 142.7, *p* = 0.0069; p < 0.05]. **C** fMLP-treated neutrophil-like dHL-60 cells adhesion on human aortic endothelial (HAEC) monolayer previously exposed to normal (NG) or high glucose (HG) for 30,120 and 360 min. The subtraction of basal and after washing the non- adhered and stained neutrophils to HAEC were quantified by ChemiDoc MP Imaging and represented as percentage over basal. A workflow of this process is shown. Graph represents the paired data regarding NG and HG. The statistical analysis showed differences on 30min treatment [n = 5, NG 0.5 h (79.58 ± 6.75) *vs* HG 0.5 h (95.46 ± 4.13); t(4) = 4.469, *p* = 0.0111; p < 0.05] and 120 min [n = 5, NG 2h (79.39 ± 10.49) *vs* HG 2h (87.65 ± 6.916); t(4) = 2.857, *p* = 0.046; p < 0.05]. Validation was also performed by fluorescence microscopy images (HAEC are shown in red, whereas dHL-60 cells, in green. Scale bar = 100 μm)

**Fig. 4:**
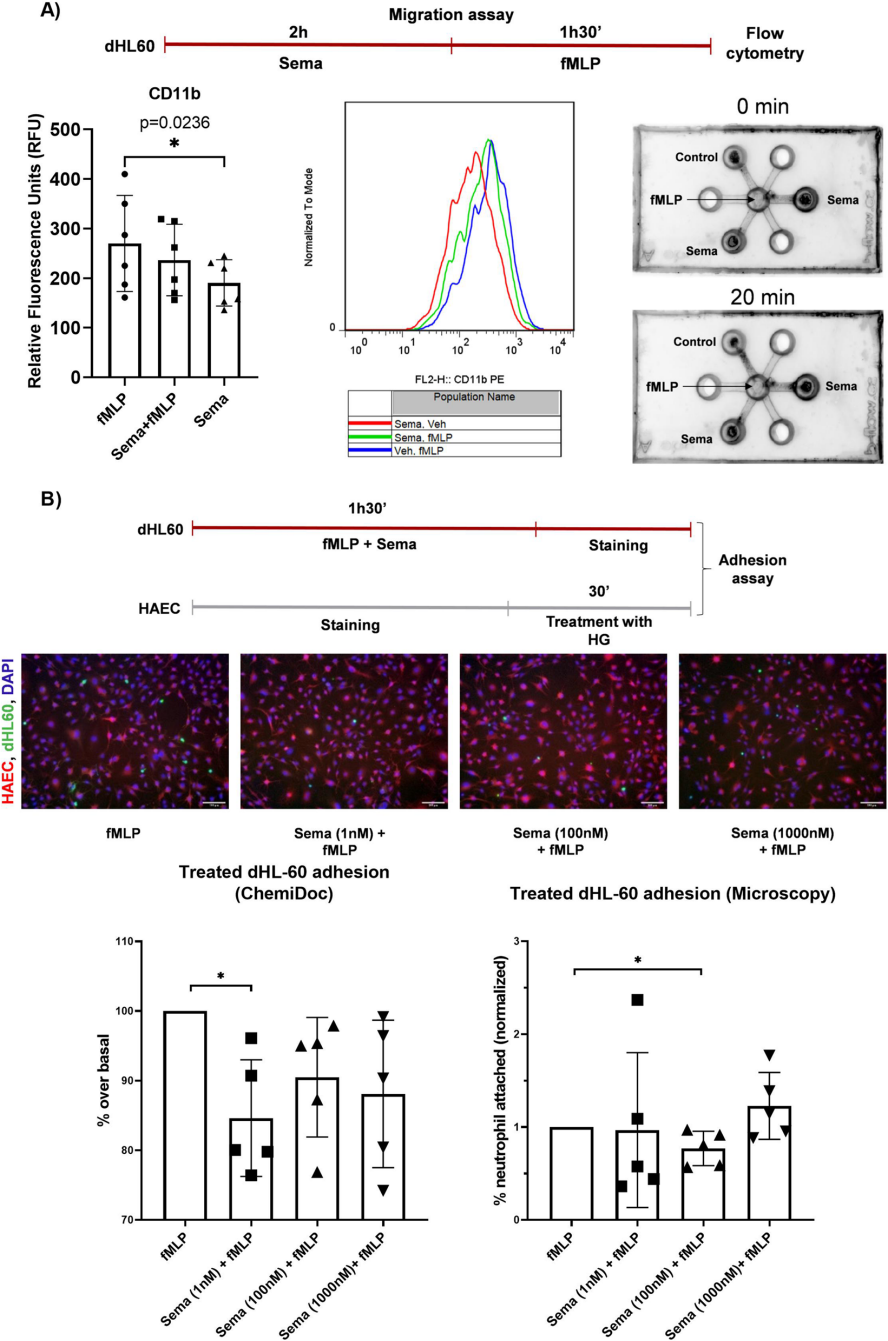
CD11b and endothelial adhesion modulation by semaglutide. **A** Work flow of the assay based on neutrophil-like dHL-60 cells treatment with semaglutide (Sema) 1 nM for 2 h and following analysis a) migration on a Sun-Chip or b) treatment with fMLP (10 μM, 90 min) and CD11b analysis by flow cytometry. Bar graph with individual data points represents the CD11b levels after treatments [n = 6; one-way ANOVA, F(2,5) = 9.817, *p* = .0236; p < 0.05; Dunett’s post-hoc fMLP (270 ± 97.04) *vs* Sema (190.5 ± 46.72), adjusted *p* value = 0.0433; p < 0.05]. Representative histogram of flow cytometry analysis or Sun-Chip migration assay. **B** Work flow of the assay based on neutrophil-like dHL-60 cells with fMLP (10 μM), with or without Sema treatment at 1 nM, 100 nM or 1000 nM, treatment for 90 min and its adhesion to aortic endothelial cells (HAEC) monolayer after high glucose (HG) treatment for 30 min. Representative fluorescence microscopy images are shown (HAEC in red, dHL-60 cells, in green. The nuclei are counterstained with DAPI. Scale bar = 100 μm). Bar graph with individual data points shows the analysis based on ChemiDoc [n = 5, Veh + fMLP (100 ± 0) *vs* Sema (1 nM) + fMLP (84.62 ± 8.37); t(4) = 4.106, *p* = .0148; p < 0.05] or microscope images [n = 5, Veh + fMLP (100 ± 0) *vs* Sema (100 nM) + fMLP (0.769 ± 0.185); t(4) = 2.780, *p* = 0.0498; p < 0.05]

The preclinical model of neutrophils (dHL-60) expressed GLP-1R and CD11b, adhesion molecule, (Fig. 3A) and had migration activity (Fig. 3B) after fMLP, chemoattractant, treatment (n = 6, Control (195 ± 45.53) *vs* fMLP (207 ± 47.24) RFU; t(5) = 3.985, p = 0.0105; p < 0.05). New method for neutrophils and endothelial adhesion assay was performed after testing the strong correlation between number of stained cells, fluorescence detection, and imaging visualization (Fig. 3B). The neutrophils with upregulated CD11b, after fMLP treatment for 90 min, were highly adhered into monolayer of pre-exposed aortic endothelial cells with HG for 30 min (n = 5, (79.58 ± 6.75) *vs* (95.46 ± 4,13); t(4) = 4.469, p = 0.0111; p < 0.05) or 120 min (n = 5, (79.39 ± 10.49) *vs* (87.65 ± 6,916); t(4) = 2.857, p = 0.046; p < 0.05) (Fig. 3C). Pre- or cotreatment with semaglutide was able to modulate the CD11b levels on neutrophils (n = 6; one-way ANOVA, F(2.5) = 9.817, p = 0.0236; p < 0.05; Dunett's multiple comparisons test fMLP (270 ± 97.04) *vs* Sema (190.5 ± 46.72) RFU, adjusted p value = 0.0433; p < 0.05) (Fig. 4A) and their aortic endothelial adhesion (Fig. 4B) induced by chemoattractant fMLP (n = 5, fMLP (100 ± 0) *vs* Sema (1 nM) + fMLP (84.62 ± 8.37)%; t(4) = 4.106, p = 0.0148; p < 0.05).

### Human markers on patients under semaglutide treatment

**Fig. 5:**
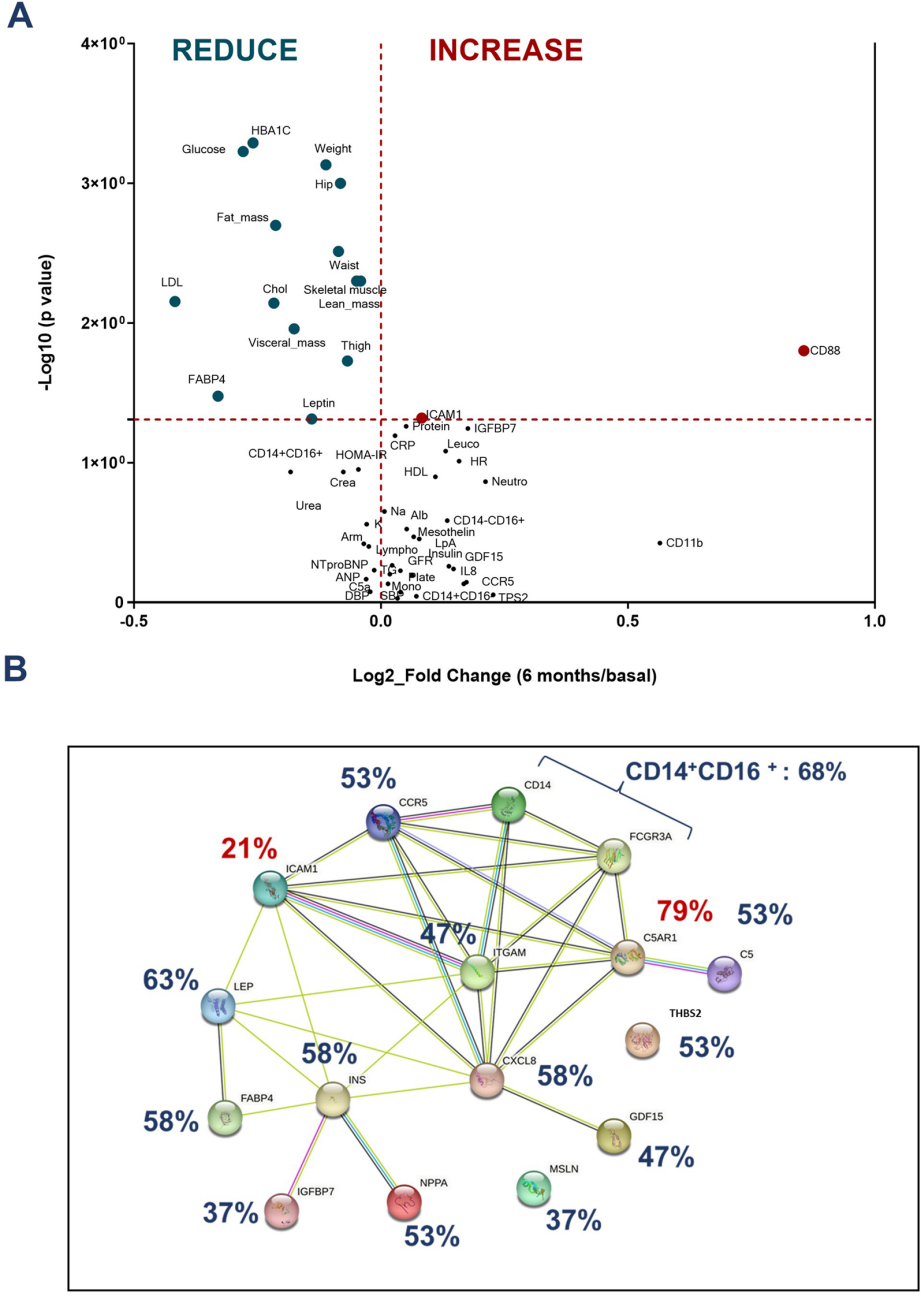
Markers levels on patients after 6 months semaglutide treatment. **A** Volcano plot represents the fold change of main markers levels on patients with cardiovascular disease after semaglutide treatment. **B** STRING Network among analysed biomarkers on treated patients with semaglutide. % of patients who reduced (blue) or increased (red) markers levels: ITGAM: CD11B; NPPA: ANP; THBS2:TSP2; C5AR1: CD88; CXCL8:IL8; FCGR3A: CD16; M2: pro-inflammatory macrophages CD14^+^CD16^+^

Between February and September 2022, we included 21 patients, average age 63 [12] years old, mainly men (81%), with significant obesity (BMI 37.4 (6.4) kg/m^2^) and high prevalence of high blood pressure (HBP), dyslipidaemia (refers to hypercholesterolemia, defined as elevation of total cholesterol and/or LDL-cholesterol or non-HDL-cholesterol in the blood) and T2DM (76, 72 and 95%, respectively) with medium duration 11.3 (9.5) years. Other CVD risk factors were also represented (atrial fibrillation (AF), HF and CAD (24, 19 and 38%, respectively)). We observed good basal therapy for CVD risk factors, most of the patients were treated with statins (91%), SGLT2i and metformin (76 and 67%, respectively) and lower use of insulin (29%). Medium left ventricle ejection fraction (LVEF) was 53% and 38% had non-preserved LVEF (≤ 50%). Ventricular hypertrophy was present in 43% and 2 severe valvulopathies were detected (mitral regurgitation and aortic stenosis), Table 1. The main important differences between included males and females were the smoker prevalence, ezetimibe intake and non-preserved LVEF (Table 1). During 6 months follow up, 1 patient died (CV event) with no other CV events. We did not observe significant differences regarding hemogram, coagulation and basic biochemistry parameters. However, there was an improvement on glucose and lipid metabolism profile. Patients reduced with statistical significance 17% of HbA1c (8.2% [2] *vs* 6.5% (0.73); p = 0.001), 14% of cholesterol levels (153 (120–189) *vs* 126 (108–141) mg/dL; p = 0.007) and 26% of LDL-Cholesterol (LDL-C) (83 (59–118) *vs* 55 [45–70] mg/dL; p = 0.007). Our results showed that 85% of included patients reduced body weight, being significant (reduction ≥ 10% of total weight) in 25%. Average of body weight reduction was 9% (104 (91–123) *vs* 96 (81–114) kg; p = 0.001). Mean reduction of waist ratio was 8.4 cms, and 8.1 cms for hip ratio. Arm and thigh ratios were also reduced in 45 and 65% patients (3.2, 4.5 cms, respectively) after 6 months treatment with semaglutide. The InBody test showed 14% fat mass reduction (41(34–58) vs 34 (28–51) kg; p = 0.002), 12% visceral fat area (216(177–248) *vs* 193 (140–247) cm^2^; p = 0.011), 3% lean mass (60 (61(50–68) vs (47–64) kg; p = 0.005) and 4% skeletal muscle (36(29–40) vs 35(27–38) kg; p = 0.002). Similar values were observed regarding NTproBNP after semaglutide treatment (290 (40–974) *vs* 215 (27–483) pg/mL). However, there was a significant reduction (21%) of adiposity marker levels FABP4 (31 (24–72) *vs* 27 (14–34) ng/mL, p = 0.033). A slight change was also observed on leptin (10%) or ICAM (5%) levels (p = 0.04). We did not observe statistical differences according monocytes phenotype but there was a 81% of CD88 levels increment on neutrophils from patients after 6 months treatment (399 (312–612) *vs* 565 (393–930) RFU, p = 0.016) (Fig. 5A).The network among different markers showed that the metabolic proteins were reduced in almost 60% of patients, the pro-inflammatory markers of monocytes or neutrophils of plasma proteins were reduced in almost 50% of patients and CD88 was upregulated in 79% of patients (Fig. 5B). Changes of markers levels on women and men are represented on Additional file 1: Table S1 and Additional file 2: Table S2, respectively.

**Table 1:** Basal clinical characteristics

	All (n = 21)	Women (n = 4)	Men (n = 17)	p
Age years old	63 (12)	66 (11)	62 (12)	0.596
Sex (Men) (%)	17 (81)			
BMI (kg/m^2^)	37.4 (6.4)	37.4 (6.4)	36.9 (4.7)	0.855
Waist (cms)	122 (13)	117 (20)	108 (17)	0.349
Hip (cms)	119 (15)	125.9 (15)	115 (16)	0.293
Arm (cms)	34.1	34.3 (6.3)	33.8 (2.9)	0.945
Smoker (%)	11 (52)	0 (0)	10 (62.5)	0.020
Alcohol (%)	3 (14)	1 (25)	2 (12.5)	0.496
HBP (%)	16 (76)	3 (75)	12 (75)	0.950
DM (%)	20 (95)	3 (75)	16 (100)	0.035
Dyslipaemia (%)	16 (72)	3 (75)	13 (81)	0.950
AF (%)	5 (24)	0 (0)	5 (31.3)	0.214
HF (%)	4 (19)	0 (0)	4 (25)	0.281
LVEF (%)	53 (9.4)	61 (0.9)	52 (9.0)	0.001
CAD (%)	8 (38)	0 (0)	7 (43.8)	0.081
CVA (%)	1 (5)	0 (0)	1 (6.3)	0.608
CKD (%)	4 (19)	0 (0)	4 (25)	0.281
OSA (%)	5 (24)	0 (0)	4 (25)	0.364
ACEi (%)	5 (24)	0 (0)	4 (25)	0.214
ARB (%)	5 (24)	1(25)	4 [25]	0.950
ARNI (%)	3 (14)	0 (0)	3 (18.8)	0.430
MRA (%)	8 (38)	0 (0)	7 (43.8)	0.081
B-B (%)	11 (52)	1 (75)	9 (56.3)	0.223
Antipl (%)	9 (43)	0 (0)	8 (50)	0.054
OAC (%)	5 (24)	0 (0)	5 (31.3)	0.241
Statins (%)	19 (91)	3 (75)	15 (93.8)	0.241
Ezetimibe (%)	12 (57)	0 (0)	11 (68.8)	0.010
Metformin (%)	14 (67)	2 (50)	11 (68.8)	0.432
SGLT2i (%)	16 (76)	3 (75)	12 (75)	0.950
Insulin (%)	6 (29)	1 (25)	4 (25)	0.867

HBP: High blood pressure; DM: diabetes mellitus type 2; AF: atrial fibrillation; HF: heart failure; CAD: coronary artery disease; CVA: cerebrovascular accident; CKD: chronic kidney disease; OSA: obstructive sleep apnea: ACEi: angiotensin-converting enzyme inhibitors: ARB: angiotensin receptor blockers; ARNI:angiotensin receptor neprylisin inhibitor; MRA: mineralocorticoid receptor antagonist; B-B: b-blockers; Antipl: antiplatelets; OAC: oral anticoagulants; SGLT2i: sodium-glucose cotransporter 2 inhibitors; BMI: body mass index; LVEF: left ventricle ejection fraction. Statistical differences between men and women are showed with p < 0.05

## Discussion

For the first time our results showed an improvement on the antiathero-thrombotic profile after semaglutide treatment based on endocrine activity of epicardial adipose tissue, modulation of neutrophils phenotype, its migration and endothelial adhesion. We can speculate that our findings can provide a potential explanation on the mechanisms behind the cardio-protective effects of semaglutide. Our results may open new avenues in the clinical use of weekly GLP-1 analogues that may go beyond the current indications. Semaglutide is a long-acting GLP-1ra that reduced 14.9% of body weight in patients with obesity or overweight without diabetes for 68 weeks treatment [23] or 13% in patients with obesity and HFpEF for 52 weeks treatment [50] Despite all GLP1-ra had demonstrated an improvement of cardiometabolic parameters (glucose, lipids, etc.…) and cardiac CT was not performed in our patients, a randomized clinical trial showed an efficient reduction of epicardial fat after semaglutide therapy [51]. This fat tissue expresses GLP1R [51], as it was also confirmed by our results, and its metabolism and endocrine activity might be modified semaglutide. The ex vivo fat tissue assay model showed insulin response on 75% of tested samples, similar results were previously published [52]. Although the increment was only significant on SAT samples. Even, the combined treatment with semaglutide improved the insulin-induced glucose consumption. However, epicardial fat from samples, with significant insulin response, were selected to carry out proteomic analysis. According to our previous results, insulin responder’s samples were represented by 75% [52]. Released exosomes by fat pads were isolated and validated with tetraspanin detection antibodies, CD63, CD81, CD9. However, only CD9 was identified, which was already described in adipose tissue-released exosomes [53]. Its levels were reduced after washing at 4ºC for 90 min. This method guaranteed a blood cleaner sample. Extracellular vesicles with inflammatory cytokines had also been detected on epicardial fat from patients with chronic AF [54]. This study had collected epicardial fat-vesicles for 9 days. However, we considered only an acute secretion of fat tissue for avoiding cells apoptosis or death. In consequence, our results have identified a few differential proteins among treatments. According insulin response on EAT, the exosomes-protein cargo analysis determined insulin pathway-related proteins after insulin treatment. However, SAT, from the selected patients, was less insulin responder. It might explain the absence of differential proteins regarding control. Semaglutide was not able to increase glucose consumption on any fat depots. Previous data had also demonstrated that GLP-1 did not increase glucose uptake in human adipocytes [55], although might modify the insulin-dependent glucose transporters levels [56]. However, after semaglutide treatment on epicardial fat, we detected gelsolin (GSN or GELS). This protein has a protector role since lower secretion levels by this fat pad are related to postoperative atrial fibrillation [57] or thrombosis or atherosclerosis process [58]. In SAT, the insulin cotreatment with semaglutide enhanced the identified exosomes-related proteins and consequently, GSN. Other identified protein was FABP4. Its levels are higher expressed on SAT than on EAT [59]. One of the main reasons is that this molecule is increased after adipogenesis induction [60] which is higher on SAT compared to EAT cells [61]. Despite higher FABP4 levels on SAT, higher inflammatory cytokines profile was described on EAT, specifically in CAD patients [62]. If GLP-1RA and insulin have a vasodilator effect [63], might also explain higher secretion of exosomes-related proteins from SAT into extratissue medium [64]. Thus, several differential proteins were identified according to this cotreatment, i.e. ANXA5 (anticoagulant protein), CO4B (complement protein), GSN (antithrombotic protein), PEDF (insulin resistance-related protein) or PRDX1 (detoxification-related protein). Although, this behaviour was not detected on EAT, where vasodilation had to be also visualized after combined treatment insulin and semaglutide. Another possible explanation might be the higher insulin resistance of the selected SAT samples that might enhance lipolysis and consequently, higher FABP4 secretion [65]. Thus, acute treatment with GLP-1 in fat tissue with insulin resistance might enhance lipolysis and consequently, increase circulating FABP4. High levels of this molecule were associated with fat mass, atherogenesis [59] and lower cardiac functional capacity [66] in patients with HF. For this reason, we also analyzed the inflammatory effects of FABP4 and its modulation by semaglutide treatment. Despite FABP4 activates neutrophils, semaglutide was able to modulate it. In fact, body fat mass, glucose and HOMA-IR were reduced after 6 months treatment with semaglutide. Consequently, less insulin resistance suggested a reduction of lipolysis and circulating FABP4 levels in patients after 6 months treatment. Previous data had demonstrated a higher CD11b mRNA expression levels in neutrophils from patients with obesity and CVD and subcutaneous fat-released molecules might be possible mediators [8]. In our population, high neutrophils-CD11b was positively associated with plasma insulin levels (r = 0.5; p < 0.05) which are related to adipose tissue resistance. In addition, FABP4 might be part of released vesicles by fat tissue with insulin resistance [67] or lipolysis consequence [55]. However, semaglutide treatment was able to modulate the neutrophils- inflammatory phenotype induced by FABP4. Moreover, plasma FABP4 levels were reduced in treated patients with semaglutide, which might be explained by a fat mass reduction. Despite of the heterogenicity of the studied population, the main advantage was that semaglutide intake was the only common factor among them. After analysing differential behaviour regarding drugs intake, we observed that FABP4 fold change was lower in those patients who were not taking angiotensin receptor blockers (ARB). After selecting only patients who were not taking ARB, our data confirmed a higher FABP4 levels reduction (p < 0.01) after semaglutide treatment. This drug improves the metabolic parameters and markers which are involved on inflammatory response. Similar mechanism might be induced and modulated after bacterial chemoattractant exposure. This is the first time that an upregulation of CD11b was observed after FABP4 treatment. GLP1-RA can activate protein kinase B (PKB) and inhibit nuclear translocation of nuclear factor-κB (NF-κB) [68]. Its p65 unit is involved in CD11b upregulation [69]. Although more mechanistic studies are needed for understanding the real associated mechanism under semaglutide effect, previous studies suggested a NF-kB dependent-pathway. Some animal models have already demonstrated that GLP-1 might modulate the CD11b expression and reduce the myocardial infarction size [18]. It might be a preventive therapy in patients with high cardiovascular risk. In fact, in those patients with high glucose and endothelial inflammation, semaglutide might modulate the neutrophils adhesion and consequently atherogenesis. Although it was not statistically significant, C5a and pro-inflammatory monocytes were reduced in 53% and 68% of patients after 6 months treatment with semaglutide, respectively. However, after studying the association with others drugs intake from patients, we observed lower C5a levels in those patients who were taking antiplatelets, which can be associated with thrombosis pathway. In this sense, C5a levels reduction was not due to semaglutide treatment alone. Contrary, we found that neutrophils CD88 is increased in 76% of patients under semaglutide treatment and was not affected by other drugs intake. This is a receptor of complement C5a, which is related to atherosclerosis [70]. However, in an inflammatory process, CD88 might be cleaved and modify the neutrophils defence and bacteria’s clearance [44]. If downregulation of CD88 is known to be associated with injury severity and is increased in neutrophils after semaglutide treatment, our results also suggest an anti-inflammatory benefit of this drug in patients with obesity and CVD.

## Conclusions

Our preclinical studies showed that semaglutide modulates the pro-inflammatory and pro-thrombotic profile on epicardial fat, neutrophils, and aortic endothelial cells. These results are allied with the reduction of fat and visceral mass, adiposity marker FABP4 levels and increment of neutrophils-CD88 in treated patients with semaglutide. These results might suggest the anti-atherothrombotic effects of this drug and new preventive and therapeutic indications of weekly GLP-1 analogues.

## Limitations

Consecutive patients were included for obtaining epicardial and subcutaneous fat and their insulin resistance was tested by “ex vivo” assays. The short time of semaglutide exposure and small piece of epicardial fat did not allow identify more proteins by proteomics study. We have included a small group of patients. Fat mass was determined by Bioimpedance and Dual X-ray Absorptiometry might be more accurate. Total visceral fat was considered. We did not perform a randomized clinical trial. All included patients have obesity and T2DM. General diet recommendations and exercise were prescribed according to clinical practice. However, any diet-based markers were registered at basal and during follow-up. Despite 5 of the included patients had morbid obesity, none of them were in a weight management program. This study observed changes of markers after being treated with semaglutide. The absence of any diet-based marker does not allow us to confirm that the body weight reduction was caused by semaglutide treatment. Although diet recommendations do not reach the 80% of success, as it was observed in our population, and several STEP trials have already demonstrated it. Genetic test was not performed for detecting familial hypercholesterolemia; however, it was not suggested in the included patients.

## A translational perspective

There is an improvement on antiathero-thrombotic profile after semaglutide treatment based on its effects on endocrine activity of epicardial adipose tissue, modulation of neutrophils phenotype and their endothelial adhesion. We can speculate that our findings can provide a potential explanation on the mechanisms behind the cardio-protective effects of semaglutide. Our results may open new avenues in the clinical use of weekly GLP-1 analogues that may go beyond the current indications.

### Supplementary Information

**Additional file 1: Table S1.** Markers on women at basal (0) and after 6 months (6m) semaglutide treatment.

**Additional file 2: Table S2.** Markers on men at 0 (0) and after 6 months (6m) semaglutide treatment.

## Data Availability

The datasets used and/or analyzed during the current study are available from the corresponding author upon reasonable request.
