# Supplementary material for: Semaglutide modulates prothrombotic and atherosclerotic mechanisms, associated with epicardial fat, neutrophils and endothelial cells network
Source: Cardiovasc Diabetol. 2024 Jan 3;23:1. doi: 10.1186/s12933-023-02096-9 (PMC10765851; doi:10.1186/s12933-023-02096-9)
Supplement: Supplementary file 1 — Additional file 1: Table S1. Markers on women at basal (0) and after 6 months (6m) semaglutide treatment. [file 12933_2023_2096_MOESM1_ESM.docx]

**Additional table 1.** Markers on women at basal (0) and after 6 months (6m) semaglutide treatment

|  | **N** | **Mean** | **SD** | **Min** | **Max** | **Percentiles** | | |
| --- | --- | --- | --- | --- | --- | --- | --- | --- |
|  |  |  |  |  |  | **25th** | **50th (Median)** | **75th** |
| Age | 4 | 66.00 | 11.43 | 50.00 | 76.00 | 54.00 | 69.00 | 75.00 |
| SBP_0 mmHg | 4 | 138.75 | 18.95 | 117.00 | 163.00 | 121.50 | 137.50 | 157.25 |
| SBP_6m | 4 | 134.00 | 18.06 | 109.00 | 151.00 | 115.25 | 138.00 | 148.75 |
| DBP_0 | 4 | 100.00 | 28.13 | 78.00 | 140.00 | 79.25 | 91.00 | 129.75 |
| DBP_6m | 4 | 84.25 | 11.24 | 75.00 | 99.00 | 75.25 | 81.50 | 96.00 |
| HR_0 | 4 | 75.25 | 7.37 | 66.00 | 84.00 | 68.25 | 75.50 | 82.00 |
| HR_6m | 4 | 74.00 | 4.97 | 68.00 | 80.00 | 69.25 | 74.00 | 78.75 |
| Weight_0 kg/m2 | 4 | 92.40 | 17.85 | 76.00 | 117.00 | 77.85 | 88.30 | 111.05 |
| Weight_6m | 4 | 84.70 | 16.19 | 70.00 | 106.00 | 71.13 | 81.40 | 101.58 |
| Waist_0 cm | 4 | 116.68 | 20.41 | 94.50 | 135.00 | 96.93 | 118.60 | 134.50 |
| Waist_6m | 4 | 106.75 | 19.75 | 86.00 | 128.00 | 88.13 | 106.50 | 125.63 |
| Hip_0 cm | 4 | 125.90 | 15.20 | 114.50 | 147.00 | 114.65 | 121.05 | 142.00 |
| Hip_6m | 4 | 118.88 | 14.20 | 101.50 | 134.00 | 104.63 | 120.00 | 132.00 |
| Arm_0 cm | 4 | 34.30 | 6.30 | 27.50 | 42.10 | 28.48 | 33.80 | 40.63 |
| Arm_6m | 4 | 30.38 | 2.50 | 27.00 | 33.00 | 27.88 | 30.75 | 32.50 |
| Thigh_0 cm | 4 | 59.53 | 10.95 | 48.20 | 74.10 | 49.98 | 57.90 | 70.70 |
| Thigh_6m | 4 | 54.13 | 6.57 | 46.50 | 62.50 | 48.13 | 53.75 | 60.50 |
| LEUC_0 x10^3^/uL | 4 | 5.78 | 1.21 | 4.09 | 6.70 | 4.50 | 6.17 | 6.68 |
| LEUC_6m | 4 | 6.36 | 0.94 | 5.24 | 7.29 | 5.42 | 6.45 | 7.20 |
| NEUTR_0 x10^3^/uL | 4 | 3.15 | 0.71 | 2.42 | 3.76 | 2.48 | 3.21 | 3.76 |
| NEUTR_6m x10^3^/uL | 4 | 3.38 | 0.67 | 2.78 | 4.08 | 2.79 | 3.33 | 4.02 |
| LYM_0 x10^3^/uL | 4 | 1.90 | 0.48 | 1.20 | 2.24 | 1.39 | 2.07 | 2.22 |
| LYM_6m | 4 | 2.11 | 0.24 | 1.79 | 2.33 | 1.86 | 2.16 | 2.31 |
| EOSIN_0 x10^3^/uL | 4 | 0.39 | 0.29 | 0.18 | 0.80 | 0.19 | 0.29 | 0.69 |
| EOSIN_6m | 4 | 0.31 | 0.18 | 0.07 | 0.45 | 0.13 | 0.36 | 0.45 |
| MONO_0 x10^3^/uL | 4 | 0.37 | 0.12 | 0.24 | 0.52 | 0.26 | 0.37 | 0.49 |
| MONO _6m | 4 | 0.40 | 0.08 | 0.31 | 0.47 | 0.32 | 0.41 | 0.47 |
| PLAT_0 x103/uL | 4 | 243.75 | 57.47 | 184.00 | 317.00 | 192.00 | 237.00 | 302.25 |
| PLAT_6m | 4 | 265.75 | 73.52 | 171.00 | 347.00 | 192.25 | 272.50 | 332.50 |
| GLUC_0 mg/dL | 4 | 138.75 | 30.92 | 103.00 | 171.00 | 108.25 | 140.50 | 167.50 |
| GLUC_6m | 4 | 121.25 | 25.75 | 89.00 | 147.00 | 95.00 | 124.50 | 144.25 |
| HBA1C_0 % | 4 | 7.45 | 1.07 | 6.00 | 8.30 | 6.33 | 7.75 | 8.28 |
| HBA1C_6m | 4 | 6.65 | 0.90 | 5.60 | 7.40 | 5.75 | 6.80 | 7.40 |
| CREA_0 mg/dL | 4 | 0.59 | 0.11 | 0.45 | 0.71 | 0.48 | 0.60 | 0.69 |
| CREA_6m | 4 | 0.64 | 0.07 | 0.54 | 0.70 | 0.57 | 0.66 | 0.70 |
| GFR_0 ml/min/1.73m^2^ | 4 | 89.60 | 0.49 | 89.00 | 90.00 | 89.10 | 89.70 | 90.00 |
| GFR_6m | 4 | 89.85 | 0.30 | 89.40 | 90.00 | 89.55 | 90.00 | 90.00 |
| Na_0 mmol/L | 4 | 140.25 | 4.27 | 134.00 | 143.00 | 135.75 | 142.00 | 143.00 |
| Na_6m | 4 | 140.75 | 2.22 | 138.00 | 143.00 | 138.50 | 141.00 | 142.75 |
| K_0 mmol/L | 4 | 4.78 | 0.26 | 4.50 | 5.00 | 4.53 | 4.80 | 5.00 |
| K_6m | 4 | 4.55 | 0.24 | 4.40 | 4.90 | 4.40 | 4.45 | 4.80 |
| UREA_0 mg/dL | 4 | 38.25 | 3.86 | 36.00 | 44.00 | 36.00 | 36.50 | 42.25 |
| UREA_6m | 4 | 39.50 | 1.73 | 38.00 | 42.00 | 38.25 | 39.00 | 41.25 |
| CHOL_0 mg/dL | 4 | 177.75 | 16.88 | 153.00 | 189.00 | 160.00 | 184.50 | 188.75 |
| CHOL_6m | 4 | 159.00 | 28.72 | 140.00 | 201.00 | 140.25 | 147.50 | 189.25 |
| HDL_0 mg/dL | 4 | 46.50 | 9.33 | 39.00 | 60.00 | 39.75 | 43.50 | 56.25 |
| HDL_6m | 4 | 53.25 | 5.06 | 49.00 | 59.00 | 49.00 | 52.50 | 58.25 |
| LDL_0 mg/dL | 4 | 106.25 | 18.79 | 83.00 | 129.00 | 88.75 | 106.50 | 123.50 |
| LDL_6m | 4 | 82.00 | 30.25 | 57.00 | 126.00 | 60.50 | 72.50 | 113.00 |
| LpA_0 | 3 | 57.00 | 42.04 | 16.00 | 100.00 | 16.00 | 55.00 | 100.00 |
| LpA_6m | 4 | 102.00 | 81.35 | 17.00 | 194.00 | 26.00 | 98.50 | 181.50 |
| TG_0 mg/dL | 4 | 125.00 | 25.07 | 101.00 | 160.00 | 104.75 | 119.50 | 150.75 |
| TG_6m | 4 | 118.75 | 42.25 | 78.00 | 170.00 | 81.25 | 113.50 | 161.50 |
| ALBU_0 mg/dL | 4 | 4.68 | 0.25 | 4.40 | 5.00 | 4.45 | 4.65 | 4.93 |
| ALBU_6m | 4 | 4.58 | 0.25 | 4.30 | 4.90 | 4.35 | 4.55 | 4.83 |
| PROT_0 | 4 | 7.08 | 0.43 | 6.80 | 7.70 | 6.80 | 6.90 | 7.53 |
| PROT_6m | 4 | 7.10 | 0.22 | 6.80 | 7.30 | 6.88 | 7.15 | 7.28 |
| FERRIT_0 | 4 | 55.25 | 17.69 | 42.00 | 80.00 | 42.25 | 49.50 | 74.00 |
| FERRIT_6m | 4 | 65.75 | 16.28 | 54.00 | 89.00 | 54.25 | 60.00 | 83.00 |
| CRP_0 | 2 | 1.25 | 0.22 | 1.09 | 1.40 | 0.82 | 1.25 | 6.30 |
| CRP_6m | 4 | 0.15 | 0.13 | 0.05 | 0.34 | 0.06 | 0.10 | 0.29 |
| NTproBNP_0 pg/mL | 4 | 115.50 | 113.87 | 24.00 | 279.00 | 31.75 | 79.50 | 235.25 |
| NTproBNP_6m | 4 | 109.00 | 137.28 | 27.00 | 314.00 | 29.50 | 47.50 | 250.00 |
| HOMA_IR_0 | 4 | 10.73 | 13.76 | 1.70 | 31.01 | 1.95 | 5.10 | 25.13 |
| HOMA_IR_6m | 4 | 11.28 | 14.26 | 1.80 | 32.10 | 1.93 | 5.60 | 26.30 |
| **NEUTR PHENOTYPE** |  |  |  |  |  |  |  |  |
| CXCR2_0 RFU | 4 | 161.50 | 58.81 | 100.00 | 240.00 | 110.25 | 153.00 | 221.25 |
| CXCR2_6m | 4 | 157.75 | 25.40 | 121.00 | 177.00 | 131.00 | 166.50 | 175.75 |
| CD11b_0 RFU | 4 | 175.23 | 105.77 | 76.80 | 292.00 | 81.38 | 166.05 | 278.25 |
| CD11b_6m | 4 | 230.65 | 318.85 | 12.60 | 702.00 | 25.45 | 104.00 | 562.50 |
| CD88_0 RFU | 4 | 397.00 | 226.82 | 141.00 | 617.00 | 174.25 | 415.00 | 601.75 |
| CD88_6m | 4 | 548.75 | 97.51 | 429.00 | 659.00 | 452.25 | 553.50 | 640.50 |
| **MONO PHENOTYPE** |  |  |  |  |  |  |  |  |
| CD14^+^CD16^-^_%_0 | 4 | 77.00 | 11.34 | 61.40 | 86.60 | 65.05 | 80.00 | 85.95 |
| CD14^+^CD16^-^_%_06 | 4 | 69.13 | 25.18 | 33.80 | 88.50 | 42.45 | 77.10 | 87.83 |
| CD14^+^CD16^+^_%_0 | 4 | 9.09 | 3.70 | 5.27 | 14.00 | 5.86 | 8.54 | 12.86 |
| CD14^+^CD16^+^_%_06 | 4 | 7.96 | 9.20 | 0.99 | 21.50 | 1.84 | 4.67 | 17.36 |
| CD14^-^CD16^+^_%_0 | 4 | 5.78 | 2.35 | 3.15 | 8.41 | 3.52 | 5.78 | 8.04 |
| CD14^-^CD16^+^_%_06 | 4 | 5.04 | 0.99 | 3.81 | 6.24 | 4.11 | 5.06 | 5.96 |
| CCR5_0 RFU | 4 | 35.78 | 53.55 | 7.02 | 116.00 | 7.07 | 10.06 | 90.23 |
| CCR5_6m | 4 | 17.08 | 6.99 | 11.50 | 26.90 | 11.80 | 14.95 | 24.48 |
| **PLASMA PROTEINS** |  |  |  |  |  |  |  |  |
| ANP_0 ng/mL | 4 | 9.92 | 1.51 | 8.19 | 11.29 | 8.42 | 10.10 | 11.24 |
| ANP_6m | 4 | 8.63 | 3.74 | 4.12 | 12.05 | 4.85 | 9.18 | 11.87 |
| FABP4_0 ng/mL | 4 | 36.92 | 16.90 | 25.91 | 62.07 | 26.59 | 29.85 | 54.32 |
| FABP4_6m | 4 | 22.67 | 9.49 | 10.55 | 32.81 | 13.06 | 23.65 | 31.28 |
| ICAM1_0 ng/mL | 4 | 313.91 | 97.37 | 225.13 | 426.79 | 228.98 | 301.86 | 410.90 |
| ICAM1_6m | 4 | 318.92 | 88.87 | 257.50 | 448.13 | 259.00 | 285.02 | 412.74 |
| IL8_0 ng/mL | 4 | 0.01 | 0.00 | 0.00 | 0.01 | 0.00 | 0.01 | 0.01 |
| IL8_6m | 4 | 0.01 | 0.01 | 0.00 | 0.02 | 0.00 | 0.01 | 0.01 |
| Leptin_0 ng/mL | 4 | 40.75 | 22.91 | 10.35 | 60.50 | 16.76 | 46.07 | 59.42 |
| Leptin_6m | 4 | 39.02 | 29.85 | 12.45 | 66.05 | 12.82 | 38.79 | 65.45 |
| Thrombospondin2_0 ng/mL | 4 | 22.20 | 7.44 | 11.33 | 28.19 | 14.60 | 24.64 | 27.35 |
| Thrombospondin2_6m | 4 | 17.18 | 8.68 | 8.70 | 26.71 | 9.29 | 16.65 | 25.60 |
| C5a_0 ng/mL | 4 | 5.30 | 1.71 | 2.99 | 7.06 | 3.57 | 5.59 | 6.76 |
| C5a_6m | 4 | 5.78 | 0.68 | 4.77 | 6.28 | 5.07 | 6.03 | 6.23 |
| GDF15_0 ng/mL | 4 | 1.29 | 0.67 | 0.59 | 2.06 | 0.67 | 1.26 | 1.96 |
| GDF15_6m | 4 | 1.23 | 0.63 | 0.55 | 1.79 | 0.62 | 1.29 | 1.78 |
| IGFBP7_0 ng/mL | 4 | 29.47 | 3.47 | 26.30 | 34.42 | 26.82 | 28.59 | 33.02 |
| IGFBP7_6m | 4 | 31.99 | 2.82 | 27.80 | 33.80 | 29.06 | 33.18 | 33.73 |
| Insulin_0 ng/mL | 4 | 1.81 | 2.44 | 0.05 | 5.40 | 0.20 | 0.89 | 4.33 |
| Insulin_6m | 4 | 2.31 | 3.46 | 0.14 | 7.42 | 0.17 | 0.84 | 5.93 |
| Mesothelin_0 ng/mL | 4 | 18.65 | 2.02 | 16.37 | 21.12 | 16.75 | 18.56 | 20.65 |
| Mesothelin_6m | 4 | 19.69 | 4.05 | 15.41 | 25.12 | 16.20 | 19.10 | 23.75 |
| **INBODY** |  |  |  |  |  |  |  |  |
| BMI _0 kg/m2 | 4 | 38.20 | 10.24 | 30.10 | 52.70 | 30.58 | 35.00 | 49.03 |
| BMI_6m | 4 | 34.78 | 8.49 | 26.90 | 46.10 | 27.65 | 33.05 | 43.63 |
| Skeletalmuscle_0 kg | 4 | 26.18 | 4.47 | 19.90 | 30.40 | 21.58 | 27.20 | 29.75 |
| Skeletalmuscle_6m | 4 | 25.30 | 3.48 | 20.60 | 28.60 | 21.68 | 26.00 | 28.23 |
| Fatfreemass_0 kg | 4 | 47.80 | 7.13 | 37.70 | 54.10 | 40.38 | 49.70 | 53.33 |
| Fatfreemass_6m | 4 | 46.63 | 5.56 | 39.10 | 51.40 | 40.78 | 48.00 | 51.10 |
| Leanmass_0 kg | 4 | 45.28 | 6.89 | 35.60 | 51.60 | 38.13 | 46.95 | 50.75 |
| Leanmass_6m | 4 | 44.03 | 5.40 | 36.80 | 48.90 | 38.38 | 45.20 | 48.50 |
| Fatmass_0 kg | 4 | 45.03 | 12.72 | 35.50 | 63.60 | 36.23 | 40.50 | 58.35 |
| Fatmass_6m | 4 | 38.88 | 12.53 | 24.80 | 55.20 | 27.70 | 37.75 | 51.18 |
| Visceralfatarea_0 cm^2^ | 4 | 224.55 | 26.84 | 196.90 | 260.90 | 201.65 | 220.20 | 251.80 |
| Visceralfatarea_6m | 4 | 195.93 | 46.91 | 133.60 | 246.80 | 149.20 | 201.65 | 236.93 |
| Metabolic rate_0 | 4 | 1402.00 | 153.52 | 1184.00 | 1537.00 | 1242.00 | 1443.50 | 1520.50 |
| Metabolic rate_6m | 4 | 1377.00 | 120.14 | 1215.00 | 1480.00 | 1250.75 | 1406.50 | 1473.75 |
| Phase angle_0 | 4 | 4.90 | 0.71 | 4.10 | 5.80 | 4.25 | 4.85 | 5.60 |
| Phase angle_6m | 4 | 4.55 | 0.89 | 3.60 | 5.70 | 3.75 | 4.45 | 5.45 |
| %_0 | 4 | 48.10 | 5.23 | 42.30 | 54.10 | 43.10 | 48.00 | 53.20 |
| %_6 | 4 | 44.75 | 7.16 | 35.20 | 51.80 | 37.35 | 46.00 | 50.90 |
