# Supplementary material for: Semaglutide modulates prothrombotic and atherosclerotic mechanisms, associated with epicardial fat, neutrophils and endothelial cells network
Source: Cardiovasc Diabetol. 2024 Jan 3;23:1. doi: 10.1186/s12933-023-02096-9 (PMC10765851; doi:10.1186/s12933-023-02096-9)
Supplement: Supplementary file 2 — Additional file 2: Table S2. Markers on men at 0 (0) and after 6 months (6m) semaglutide treatment. [file 12933_2023_2096_MOESM2_ESM.docx]

**Additional table 2.** Markers on men at 0 (0) and after 6 months (6m) semaglutide treatment

|  | **N** | **Mean** | **SD** | **Min** | **Max** | **Percentiles** | | |  |
| --- | --- | --- | --- | --- | --- | --- | --- | --- | --- |
|  |  |  |  |  |  | **25th** | **50th (Median)** | **75th** | ***p*** |
| Age | 16 | 62.94 | 12.26 | 42.00 | 79.00 | 56.00 | 64.00 | 72.25 |  |
| SBP_0 mmHg | 16 | 131.00 | 16.51 | 110.00 | 173.00 | 120.50 | 126.00 | 142.00 |  |
| SBP_6m | 16 | 137.50 | 17.63 | 114.00 | 172.00 | 118.50 | 139.00 | 148.25 |  |
| DBP_0 | 16 | 77.88 | 6.50 | 63.00 | 86.00 | 74.00 | 77.50 | 85.00 |  |
| DBP_6m | 16 | 82.25 | 10.79 | 66.00 | 109.00 | 75.50 | 80.00 | 89.25 |  |
| HR_0 | 16 | 71.13 | 10.32 | 55.00 | 90.00 | 63.25 | 72.00 | 78.75 |  |
| HR_6m | 16 | 77.00 | 8.34 | 63.00 | 94.00 | 73.00 | 76.50 | 81.50 |  |
| Weight_0 kg/m^2^ | 16 | 107.89 | 17.03 | 84.40 | 139.50 | 93.13 | 105.25 | 123.58 | 0.003 |
| Weight_6m | 16 | 100.45 | 19.82 | 65.50 | 139.20 | 86.13 | 97.80 | 116.40 |  |
| Waist_0 cm | 14 | 122.63 | 10.53 | 107.50 | 138.00 | 112.88 | 121.00 | 131.50 | 0.016 |
| Waist_6m | 16 | 116.13 | 15.89 | 90.50 | 141.50 | 101.50 | 118.00 | 127.88 |  |
| Hip_0 cm | 14 | 115.18 | 14.60 | 99.00 | 141.00 | 102.90 | 108.20 | 130.25 | 0.003 |
| Hip_6m | 16 | 107.88 | 13.13 | 89.50 | 135.00 | 100.00 | 105.50 | 119.50 |  |
| Arm_0 cm | 14 | 33.80 | 2.93 | 29.50 | 39.50 | 31.48 | 34.50 | 36.03 |  |
| Arm_6m | 16 | 33.81 | 3.80 | 28.00 | 41.50 | 30.25 | 34.00 | 36.63 |  |
| Thigh_0 cm | 14 | 56.92 | 6.26 | 49.20 | 71.30 | 52.23 | 55.65 | 61.63 |  |
| Thigh_6m | 16 | 55.11 | 7.41 | 42.50 | 73.20 | 50.50 | 54.00 | 59.88 |  |
| LEUC_0 x10^3^/uL | 16 | 7.17 | 1.82 | 3.42 | 11.38 | 5.98 | 7.05 | 7.91 |  |
| LEUC_6m | 16 | 7.64 | 2.27 | 4.08 | 12.61 | 6.04 | 7.29 | 8.68 |  |
| NEUTR_0 x10^3^/uL | 16 | 4.54 | 1.38 | 1.97 | 7.63 | 3.83 | 4.53 | 5.15 |  |
| NEUTR_6m x10^3^/uL | 16 | 5.02 | 1.58 | 2.62 | 8.75 | 4.20 | 4.86 | 5.54 |  |
| LYM_0 x10^3^/uL | 15 | 1.82 | 0.53 | 1.11 | 2.75 | 1.42 | 1.78 | 2.38 |  |
| LYM_6m | 16 | 1.87 | 0.96 | 0.34 | 4.32 | 1.30 | 1.82 | 2.20 |  |
| EOSIN_0 x10^3^/uL | 16 | 0.24 | 0.23 | 0.00 | 0.80 | 0.08 | 0.13 | 0.40 |  |
| EOSIN_6m | 16 | 0.41 | 0.74 | 0.00 | 2.80 | 0.07 | 0.16 | 0.27 |  |
| MONO_0 x10^3^/uL | 16 | 0.45 | 0.17 | 0.21 | 0.94 | 0.38 | 0.42 | 0.55 |  |
| Mono_6m | 16 | 0.45 | 0.16 | 0.22 | 0.86 | 0.33 | 0.45 | 0.54 |  |
| PLAT_0 x10^3^/uL | 16 | 241.00 | 78.19 | 108.00 | 429.00 | 197.00 | 242.50 | 287.00 |  |
| PLAT_6m | 16 | 228.00 | 60.37 | 132.00 | 336.00 | 191.75 | 224.00 | 276.00 |  |
| GLUC_0 mg/dL | 16 | 161.06 | 56.29 | 93.00 | 278.00 | 114.50 | 143.50 | 204.50 | 0.003 |
| GLUC_6m | 16 | 123.38 | 33.46 | 85.00 | 193.00 | 94.50 | 119.00 | 132.50 |  |
| HBA1C_0 % | 16 | 8.31 | 2.06 | 6.30 | 12.50 | 6.55 | 7.60 | 9.28 | 0.002 |
| HBA1C_6m | 16 | 6.53 | 0.73 | 5.70 | 8.00 | 6.00 | 6.20 | 7.10 |  |
| CREA_0 mg/dL | 16 | 1.69 | 2.23 | 0.51 | 9.91 | 0.89 | 1.01 | 1.63 |  |
| CREA_6m | 16 | 1.08 | 0.39 | 0.63 | 2.11 | 0.77 | 1.05 | 1.24 |  |
| GFR_0 ml/min/1.73m^2^ | 16 | 72.24 | 21.45 | 34.90 | 90.00 | 55.18 | 84.50 | 90.00 |  |
| GFR_6m | 16 | 72.64 | 18.51 | 36.00 | 90.00 | 57.30 | 73.20 | 90.00 |  |
| Na_0 mmol/L | 16 | 140.69 | 2.50 | 135.00 | 145.00 | 139.00 | 141.00 | 142.00 |  |
| Na_6m | 16 | 141.38 | 1.54 | 138.00 | 144.00 | 140.25 | 142.00 | 142.00 |  |
| K_0 mmol/L | 15 | 4.57 | 0.46 | 3.90 | 5.70 | 4.20 | 4.50 | 4.90 |  |
| K_6m | 15 | 4.53 | 0.55 | 3.40 | 5.60 | 4.30 | 4.50 | 4.90 |  |
| UREA_0 mg/dL | 16 | 61.63 | 29.99 | 31.00 | 139.00 | 39.50 | 50.00 | 78.25 |  |
| UREA_6m | 16 | 55.31 | 18.71 | 29.00 | 99.00 | 41.00 | 56.00 | 66.75 |  |
| CHOL_0 mg/dL | 16 | 152.31 | 51.32 | 99.00 | 261.00 | 116.25 | 135.00 | 180.50 | 0.017 |
| CHOL_6m | 16 | 120.88 | 20.71 | 85.00 | 157.00 | 106.00 | 123.00 | 135.25 |  |
| HDL_0 mg/dL | 16 | 33.75 | 8.18 | 24.00 | 51.00 | 27.25 | 31.50 | 36.75 |  |
| HDL_6m | 16 | 36.00 | 6.50 | 26.00 | 48.00 | 29.00 | 37.00 | 40.75 |  |
| LDL_0 mg/dL | 16 | 83.19 | 41.32 | 36.00 | 176.00 | 57.25 | 69.50 | 97.00 | 0.02 |
| LDL_6m | 16 | 53.56 | 22.05 | 1.00 | 99.00 | 43.50 | 53.00 | 61.00 |  |
| LpA_0 | 16 | 105.48 | 123.61 | 3.00 | 480.00 | 18.70 | 68.50 | 155.50 |  |
| LpA_6m | 14 | 99.50 | 112.14 | 3.00 | 377.00 | 18.75 | 65.00 | 153.50 |  |
| TG_0 mg/dL | 16 | 176.94 | 82.47 | 43.00 | 317.00 | 101.00 | 189.50 | 245.75 |  |
| TG_6m | 16 | 156.94 | 95.21 | 49.00 | 391.00 | 89.50 | 121.50 | 212.00 |  |
| ALBU_0 mg/dL | 16 | 4.19 | 0.35 | 3.50 | 4.80 | 4.00 | 4.15 | 4.48 |  |
| ALBU_6m | 16 | 4.39 | 0.27 | 4.00 | 5.00 | 4.20 | 4.30 | 4.58 |  |
| PROT_0 | 16 | 6.40 | 0.44 | 5.70 | 7.30 | 6.10 | 6.25 | 6.60 |  |
| PROT_6m | 16 | 6.66 | 0.31 | 6.30 | 7.30 | 6.40 | 6.60 | 6.88 |  |
| FERRIT_0 | 16 | 116.19 | 118.40 | 5.00 | 362.00 | 29.75 | 65.00 | 196.25 |  |
| FERRIT_6m | 16 | 112.25 | 80.45 | 23.00 | 351.00 | 47.00 | 105.00 | 139.50 |  |
| CRP_0 | 12 | 1.99 | 3.47 | 0.10 | 12.71 | 0.28 | 0.98 | 2.14 |  |
| CRP_6m | 16 | 0.68 | 0.83 | 0.05 | 2.81 | 0.06 | 0.38 | 1.27 |  |
| NTproBNP_0 pg/mL | 16 | 623.31 | 766.01 | 10.00 | 2630.00 | 30.00 | 377.50 | 1075.75 |  |
| NTproBNP_6m | 16 | 681.00 | 1318.41 | 10.00 | 5080.00 | 26.50 | 314.00 | 494.75 |  |
| HOMA_IR_0 | 11 | 6.05 | 3.48 | 3.00 | 12.70 | 3.30 | 5.00 | 7.30 | 0.041 |
| HOMA_IR_6m | 14 | 3.99 | 2.72 | 0.80 | 11.20 | 2.03 | 3.35 | 5.23 |  |
| **NEUTR PHENOTYPE** |  |  |  |  |  |  |  |  |  |
| CXCR2_0 RFU | 15 | 127.97 | 36.48 | 76.00 | 181.00 | 85.80 | 135.00 | 158.00 |  |
| CXCR2_6m | 16 | 128.59 | 45.21 | 58.50 | 204.00 | 85.65 | 141.00 | 154.50 |  |
| CD11b_0 RFU | 15 | 67.87 | 41.42 | 28.70 | 188.00 | 44.30 | 55.70 | 69.00 |  |
| CD11b_6m | 16 | 96.70 | 87.56 | 10.30 | 319.00 | 23.13 | 85.40 | 121.50 |  |
| CD88_0 RFU | 15 | 436.33 | 150.30 | 224.00 | 703.00 | 327.00 | 397.00 | 598.00 | 0.027 |
| CD88_6m | 16 | 668.63 | 353.61 | 221.00 | 1419.00 | 371.25 | 571.00 | 947.75 |  |
| **MONO PHENOTYPE** |  |  |  |  |  |  |  |  |  |
| CD14^+^CD16^-^_%_0 | 15 | 67.12 | 16.26 | 40.40 | 88.80 | 55.30 | 63.80 | 84.80 |  |
| CD14^+^CD16^-^_%_6m | 16 | 65.94 | 20.05 | 33.30 | 90.00 | 45.60 | 73.45 | 80.63 |  |
| CD14^+^CD16^+^_%_0 | 15 | 6.67 | 4.30 | 1.80 | 15.80 | 3.50 | 5.56 | 9.13 |  |
| CD14^+^CD16^+^_%_6m | 16 | 4.98 | 4.20 | 1.12 | 15.20 | 2.09 | 3.28 | 6.65 |  |
| CD14^-^CD16^+^_%_0 | 15 | 8.05 | 5.79 | 1.17 | 24.70 | 4.92 | 6.98 | 9.03 |  |
| CD14^-^CD16^+^_%_6m | 16 | 7.00 | 4.24 | 1.06 | 16.60 | 4.35 | 5.79 | 9.24 |  |
| CCR5_0 | 15 | 9.92 | 3.40 | 4.98 | 15.80 | 7.06 | 9.29 | 13.10 |  |
| CCR5_6m | 16 | 9.55 | 7.63 | 3.99 | 37.50 | 6.51 | 8.07 | 9.16 |  |
| **PLASMA PROTEINS** |  |  |  |  |  |  |  |  |  |
| ANP_0 ng/mL | 16 | 15.56 | 8.47 | 6.28 | 42.18 | 9.59 | 15.21 | 18.34 |  |
| ANP_6m | 16 | 16.35 | 13.74 | 5.61 | 64.20 | 8.07 | 13.50 | 19.46 |  |
| FABP4_0 ng/mL | 16 | 61.39 | 65.42 | 5.22 | 256.96 | 20.88 | 33.60 | 104.01 | 0.07 |
| FABP4_6m | 16 | 32.75 | 23.81 | 10.18 | 101.36 | 14.32 | 29.08 | 44.16 |  |
| ICAM1_0 ng/mL | 16 | 415.00 | 195.98 | 205.00 | 904.66 | 267.54 | 373.41 | 473.92 |  |
| ICAM1_6m | 16 | 438.46 | 201.22 | 163.50 | 917.89 | 315.41 | 377.77 | 515.38 |  |
| IL8_0 ng/mL | 16 | 0.01 | 0.00 | 0.00 | 0.01 | 0.00 | 0.01 | 0.01 |  |
| IL8_6m | 16 | 0.01 | 0.00 | 0.00 | 0.02 | 0.00 | 0.00 | 0.01 |  |
| Leptin_0 ng/mL | 16 | 22.03 | 21.99 | 4.04 | 91.06 | 8.91 | 12.33 | 32.34 |  |
| Leptin_6m | 16 | 18.42 | 26.49 | 3.33 | 108.83 | 5.79 | 10.26 | 16.83 |  |
| Thrombospondin2_0 ng/mL | 16 | 12.99 | 6.86 | 4.14 | 23.72 | 5.54 | 13.47 | 18.55 |  |
| Thrombospondin2_6m | 16 | 16.37 | 13.73 | 2.91 | 59.61 | 7.06 | 14.09 | 20.94 |  |
| C5a_0 ng/mL | 16 | 6.27 | 2.40 | 3.19 | 11.67 | 4.34 | 6.12 | 7.49 |  |
| C5a_6m | 16 | 5.94 | 2.11 | 2.46 | 10.28 | 4.43 | 5.89 | 7.40 |  |
| GDF15_0 ng/mL | 16 | 2.09 | 1.34 | 0.76 | 5.20 | 1.06 | 1.57 | 2.80 |  |
| GDF15_6m | 16 | 2.20 | 1.27 | 0.97 | 5.74 | 1.27 | 1.71 | 2.44 |  |
| IGFBP7_0 ng/mL | 16 | 26.37 | 10.08 | 8.34 | 54.87 | 22.28 | 24.82 | 30.96 |  |
| IGFBP7_6m | 16 | 29.15 | 11.60 | 12.28 | 59.79 | 21.37 | 27.77 | 32.64 |  |
| Insulin_0 ng/mL | 16 | 1.18 | 1.22 | 0.21 | 5.00 | 0.39 | 0.71 | 1.58 |  |
| Insulin_6m | 16 | 0.89 | 0.70 | 0.12 | 2.85 | 0.41 | 0.72 | 1.13 |  |
| Mesothelin_0 ng/mL | 16 | 24.95 | 11.71 | 8.52 | 46.72 | 15.18 | 22.07 | 33.69 |  |
| Mesothelin_6m | 16 | 25.77 | 11.53 | 11.91 | 46.58 | 13.81 | 23.09 | 37.58 |  |
| **INBODY** |  |  |  |  |  |  |  |  |  |
| BMI _0 kg/m2 | 16 | 36.98 | 4.67 | 29.70 | 46.90 | 33.58 | 35.15 | 41.40 | 0.001 |
| BMI_6m | 15 | 34.53 | 5.97 | 25.60 | 46.80 | 31.10 | 33.00 | 40.40 |  |
| Skeletalmuscle_0 | 16 | 36.11 | 5.55 | 24.60 | 45.90 | 32.98 | 36.25 | 40.43 | 0.001 |
| Skeletalmuscle_6m | 15 | 34.84 | 5.79 | 24.40 | 43.70 | 29.80 | 35.50 | 40.00 |  |
| Fatfreemass_0 | 16 | 64.83 | 9.07 | 46.10 | 79.10 | 59.43 | 65.45 | 71.78 | 0.007 |
| Fatfreemass_6m | 15 | 63.08 | 9.39 | 45.90 | 75.80 | 55.20 | 65.40 | 71.50 |  |
| Leanmass_0 | 16 | 61.33 | 8.49 | 43.70 | 74.50 | 56.28 | 61.85 | 67.83 | 0.004 |
| Leanmass_6m | 15 | 59.55 | 8.85 | 43.40 | 71.40 | 51.70 | 61.88 | 67.50 |  |
| Fatmass_0 | 16 | 43.21 | 13.27 | 24.30 | 69.50 | 32.58 | 40.30 | 56.18 | 0.003 |
| Fatmass_6m | 15 | 38.16 | 15.43 | 18.80 | 66.90 | 28.00 | 33.20 | 51.10 |  |
| Visceralfatarea_0 | 16 | 204.18 | 51.76 | 123.70 | 298.90 | 164.03 | 202.70 | 234.08 | 0.014 |
| Visceralfatarea_6m | 15 | 186.95 | 70.67 | 89.40 | 291.40 | 139.70 | 178.40 | 251.70 |  |
| Metabolic rate_0 | 16 | 1768.44 | 195.24 | 1366.00 | 2080.00 | 1654.75 | 1775.00 | 1921.00 | 0.010 |
| Metabolic rate_6m | 15 | 1732.67 | 202.88 | 1361.00 | 2006.00 | 1563.00 | 1782.00 | 1914.00 |  |
| Phage angle _0 | 16 | 5.23 | 0.91 | 4.10 | 7.50 | 4.28 | 5.20 | 5.78 | 0.010 |
| Phage angle_6m | 15 | 4.89 | 0.90 | 3.90 | 7.20 | 4.10 | 5.00 | 5.20 |  |
| %_0 | 16 | 39.43 | 7.20 | 25.50 | 50.60 | 34.28 | 40.55 | 43.95 | 0.009 |
| %_6 | 15 | 36.57 | 8.83 | 20.30 | 48.70 | 29.10 | 38.30 | 43.90 |  |
